# Metabolite patterns link diet, obesity, and type 2 diabetes in a Hispanic population

**DOI:** 10.1007/s11306-021-01835-x

**Published:** 2021-09-22

**Authors:** Laurence D. Parnell, Sabrina E. Noel, Shilpa N. Bhupathiraju, Caren E. Smith, Danielle E. Haslam, Xiyuang Zhang, Katherine L. Tucker, Jose M. Ordovas, Chao-Qiang Lai

**Affiliations:** 1 USDA Agricultural Research Service, Nutrition and Genomics Laboratory, JM-USDA Human Nutrition Research Center on Aging at Tufts University, Boston, MA USA; 2 Department of Biomedical and Nutritional Sciences, University of Massachusetts Lowell, Lowell, MA USA; 3 Channing Division of Network Medicine, Harvard Medical School and Brigham and Women’s Hospital, Boston, MA USA; 4 Department of Nutrition, Harvard T.H. Chan School of Public Health, Harvard University, Boston, MA USA; 5 Nutrition and Genomics Laboratory, JM-USDA Human Nutrition Research Center On Aging at Tufts University, Boston, MA USA; 6 IMDEA Food Institute, CEI UAM + CSIC, Madrid, Spain; 7 Centro Nacional de Investigaciones Cardiovasculares (CNIC), Madrid, Spain

**Keywords:** Metabolomics, Molecular biomarkers, Obesity, Type 2 diabetes, Diet

## Abstract

**Introduction:**

Obesity is a precursor of type 2 diabetes (T2D).

**Objectives:**

Our aim was to identify metabolic signatures of T2D and dietary factors unique to obesity.

**Methods:**

We examined a subsample of the Boston Puerto Rican Health Study (BPRHS) population with a high prevalence of obesity and T2D at baseline (n = 806) and participants (without T2D at baseline) at 5-year follow-up (n = 412). We determined differences in metabolite profiles between T2D and non-T2D participants of the whole sample and according to abdominal obesity status. Enrichment analysis was performed to identify metabolic pathways that were over-represented by metabolites that differed between T2D and non-T2D participants. T2D-associated metabolites unique to obesity were examined for correlation with dietary food groups to understand metabolic links between dietary intake and T2D risk. False Discovery Rate method was used to correct for multiple testing.

**Results:**

Of 526 targeted metabolites, 179 differed between T2D and non-T2D in the whole sample, 64 in non-obese participants and 120 unique to participants with abdominal obesity. Twenty-four of 120 metabolites were replicated and were associated with T2D incidence at 5-year follow-up. Enrichment analysis pointed to three metabolic pathways that were overrepresented in obesity-associated T2D: phosphatidylethanolamine (PE), long-chain fatty acids, and glutamate metabolism. Elevated intakes of three food groups, energy-dense takeout food, dairy intake and sugar-sweetened beverages, associated with 13 metabolites represented by the three pathways.

**Conclusion:**

Metabolic signatures of lipid and glutamate metabolism link obesity to T2D, in parallel with increased intake of dairy and sugar-sweetened beverages, thereby providing insight into the relationship between dietary habits and T2D risk.

**Supplementary Information:**

The online version contains supplementary material available at 10.1007/s11306-021-01835-x.

## Introduction

Obesity, the most common metabolic disease globally and in the US, is a precursor of type 2 diabetes (T2D) (Abdullah et al., 2011; Kodama et al., 2014). Individuals with obesity, particularly those with central adiposity, have an increased risk of developing T2D, compared to those with normal weight (Serrano Rios, 1998; Smith, 2015). Obesity is both a consequence and driver of metabolic imbalances and dysfunctional metabolic processes. However, the metabolic processes that underlie the pathogenesis of T2D are unclear. Additionally, how dietary intake contributes to and accelerates such process remains to be determined.

It has been established rather unequivocally that there are strong links between lifestyle factors and risk and incidence of type 2 diabetes (Magkos et al., 2020), particularly dietary intakes (Toi et al., 2020). Important dietary factors that have been linked to T2D risk include fat (Rice Bradley, 2018), carbohydrate (Sainsbury et al., 2018), proteins (Zhao et al., 2019), alcohol use (Han, 2020; Hirst et al., 2017). Given the habitual nature of dietary factors, their influence on metabolic function can be profound. For example, increased intake of sugar-sweetened beverage has been linked to elevated occurrence of T2D (Malik et al., 2010). Similarly, low-fat dairy intake is associated with reduced risk of T2D (Mitri et al., 2019). However, the metabolic links between dietary intake and T2D risk remain to be explored.

We hypothesized that the metabolic disruptions leading to the development of T2D may be different among obese individuals compared to non-obese individuals and this may be reflected by plasma metabolite measures. Extensive data on blood metabolites, clinical parameters, and dietary intakes support further investigations to characterize the bioprocesses involved in obesity-driven T2D in comparison to T2D in a set of non-obese individuals (Roden & Shulman, 2019). Hispanic populations in the USA have experienced severe health disparity (Tucker et al., 2010). Characterization of the metabolic mechanisms underlying such health disparity in metabolic diseases, like obesity and T2D, is still lacking. The Boston Puerto Rican Health Study (BPRHS) is well-positioned to support analyses examining the relationships between adiposity and T2D among Hispanics with a high prevalence of obesity and T2D (Tucker et al., 2010). Assessment of incident T2D at the 5-year follow-up of the BPRHS provides the opportunity to test longitudinal associations with T2D. Although adiposity, as measured by waist circumference, and occurrence of T2D are highly correlated, not all obese individuals develop T2D and not all individuals with T2D were obese before diagnosis (Boden, 1998). Thus, the BPRHS cohort offers a special opportunity to uncover the bioprocesses involved in these two distinct paths to T2D development.

## Methods

### Study population

BPRHS is a longitudinal cohort study designed to investigate the relationship between stress, nutrition, and health outcomes, like metabolic diseases, in Puerto Ricans living in the Greater Boston area (Tucker et al., 2010). Visits were conducted from 2004 to 2015 at three time points: baseline, 2-years, and 5-years. Detailed recruitment and data collection methods were described previously (Noel et al., 2009; Tucker et al., 2010). Of the 1,504 self-identified Puerto Rican adults, aged 45 to 75 years, 1311 had complete dietary, clinical, and biochemistry measures. Plasma samples from 817 participants were sent to Metabolon Inc. (Morrisville, NC, USA) for metabolomics analysis. Among these participants, 415 did were not diagnosed with T2D at baseline and completed 5-year follow-up visit, of which 66 developed T2D during the follow-up period.

#### Obesity and type 2 diabetes assessment

In this study, as waist circumference is a stronger indicator than BMI for T2D risk (see section 3), obese participants (group) were defined as those with waist ≥ 102 cm in men, ≥ 88 cm in women, whereas non-obese participants (group) were those with waist < 102 cm in men, < 88 cm in women. Type 2 diabetes: T2D is defined as either FPG ≥ 126 mg/dL, or use of hypoglycemic agents (self-reported). Clinical examinations for obesity and T2D were conducted both at baseline and 5-year follow-up.

#### Physical activity and education score

Physical activity was measured using a modified version of the Harvard Alumni survey Paffenbarger questionnaire (Paffenbarger et al., 1993). A physical activity score was calculated according to the weighted sum of hours spent on various activities during a 24-h period (Paffenbarger et al., 1993). Education score was calculated based on education level: no schooling or less than 5th grade, 5^th^–8th grade, 9^th^–12th grade, college degree, and some graduate school (Tucker et al., 2010).

#### Dietary assessment

A food frequency questionnaire (FFQ) adapted and validated for use in this population of Hispanic adults was used to assess usual dietary intake (Tucker et al., 1998). Based on similarity of key nutrient content, 126 types of foods were categorized into 34 food groups (Noel et al., 2009). For example, pizza (servings) included all types of pizza/Mexican/Other take-out food (eggroll, dumpling, turnover, empanadas, tamale, enchiladas, quesadilla, burrito, taco with beef and cheese), Soups (broth or bouillon in servings) included covered bean (pea, lentil) soup, homemade soup, ramen noodle, chicken noodle, vegetable beef or chicken soup. Soft drinks (SoftDrinks in servings) comprised of regular and diet soft drinks, fruit drinks, sweetened energy drinks and tea intake were assessed together as sweetened beverages. These include (1) regular cola and caffeine free cola; (2) carbonated drinks with added sugar; (3) juice or flavored drink, fruit-flavored nectar (peach, pear, mango, lemonade) and fruit punch, but excluding 100% fruit juice; (3) other flavored drinks with vitamins and added sugar; (4) purchased pre-sweetened ready-to-drink tea; (5) diet cola drinks and diet non-cola drinks. Total soft drink intake was calculated and converted to servings per day.

### Metabolomic profiling

Plasma samples were collected and stored at − 80 °C at baseline from all recruited participants of BPRHS. Metabolomic analysis was conducted once on 806 plasma samples collected at baseline by Metabolon Inc. (Evans et al., 2009). Briefly, metabolomic analyses were performed using ultrahigh- performance liquid chromatography-tandem mass spectroscopy after proteins were extracted from the plasma. Metabolites were detected and qualified by measuring the area under the curve of the peaks with reference to a library of over 4500 purified standards for retention time/index, mass-to-charge ratio, and chromatographic data. The measures of each metabolite were normalized across all samples and validated by the Metabolon Inc. (Wulff & Mitchell, 2018). In total, there were 526 targeted metabolites available and included in this study. Among these, 432 metabolites were designated to 54 metabolic pathways that included at least three metabolites in each pathway (Zhou et al., 2020).

### Statistical analysis

All statistical analyses were conducted using SAS 9.4 (SAS Inc.), R, or SVS 8.7 (GoldenHelix Inc). Missing data was omitted for all statistical analyses.

#### Associations between obesity, metabolites, dietary intake, and T2D

To determine the association between obesity and T2D, a logistic regression was used with T2D as the dependent variable and waist or BMI as predictors, controlling for sex, age, education, smoking, alcohol use, and physical activity. This was modeled as T2D = waist or BMI + covariates using Proc Logistic with a link function in SAS 9.4. To identify metabolites that are associated with T2D, a similar regression model was used with T2D as the dependent variable and the plasma concentration of each metabolite as an independent variable, controlling for sex, age, physical activity, education, smoking, alcohol use, and total energy intake. This was modeled as T2D = metabolite + covariates. The same model and analyses were applied to the baseline and 5-year follow-up. A False Discovery Rate using the Q-value method (Storey, 2002) was used to correct for multiple testing. To identify food groups that are associated with identified key metabolites, a linear regression model: metabolite = food group + covariates, was used with metabolites as dependent variables and food group servings as a predictor while controlling for age, sex, alcohol use, smoking, education, physical activity, and total energy intake.

#### Confirmation of associations between plasma metabolites and T2D incidence at 5-year follow-up

After identifying metabolites that were unique to obesity were associated with T2D at baseline, we further confirmed if the identified 120 metabolites were enriched among the metabolites that were associated with T2D incidence during the 5-year follow-up. With a linear regression model, the identified 120 metabolites were tested for their associations with T2D incidence at 5-year follow-up among those non-T2D participants (n = 412) at baseline. Then enrichment analysis was conducted based on identified significant association to identify over-representative pathway.

#### Metabolic pathway enrichment analysis

To determine if metabolites that are associated with T2D were enriched for any given metabolic pathway, enrichment analysis was conducted (Mangano et al., 2021; Zhou et al., 2020). All targeted metabolites were organized into metabolic pathways based on the annotation database of Metabolon Inc., and only pathways that contained three or more metabolites were included in this analysis. For each pathway, a Z score was calculated as [r – n (R/N)] /√{n (R/N)[1-(R/N)][1-(n-1)/(N-1)]}, where N is the total number of metabolites assigned into this metabolic pathway, n is the total number of metabolites measured in a specific pathway, R is the total number of metabolites that were significantly identified in the metabolic signature analysis as described above, r is the number of R metabolites that was identified as significant in a specific pathway. P values were derived from Z scores assuming a normal distribution and were two-sided. False Discovery Rate based on the Benjamini and Hochberg method (Benjamini, 1995) was used to correct for multiple testing.

#### Compound classification and enrichment

With identified statistically significantly different compounds, each unique to either the obese group (n = 120) or non-obese group (n = 12), we sought to conduct enrichment analyses as these inform which biological functions are involved in T2D under different obesity conditions. These analyses were performed in two different ways—functional categorization (e.g., pathway and bioprocess enrichment) and compound classification. This latter approach assesses enrichment of ontology or classification terms and is useful when identified metabolites are not assigned to pathways. The two- and three-tier hierarchical systems (Sumner et al., 2007) for classifying metabolites from Metabolon and LipidMaps, respectively were used.

In addition, these two sets of compounds were analyzed with the Reactome and Mbrole 2.0 tools (Fabregat et al., 2018; Lopez-Ibanez et al., 2016). These algorithms identify biochemical physiological pathways, bioprocesses and chemical classifications. This analysis, performed under the default parameters, yielded P-values of significant enrichment that were corrected by FDR (Benjamini, 1995) within each tool itself.

## Results

### BPRHS study population

The obese and non-obese groups were defined based on waist circumference (cm) for its strong association with risk of T2D (Smith, 2015). Among 315 participants with T2D, 79% (n = 249) showed central obesity, and 92% (n = 290) were obese or overweight (BMI ≥ 25). We examined the differences in general characteristics according to obese and non-obese groups (Table 1). Participants in the obese group were mainly women and had a significantly higher BMI and waist circumference, but no differences in age were observed. For lifestyle and behaviors, the obese group tended to drink less alcohol and smoke less, but showed no differences in physical activity and total energy intake were observed.

**Table 1:** General characteristics of participants with metabolome profile in BPRHS at the baseline

		Total (n = 806)	Non-obese (n = 221)	Obese (n = 585)
Age (SD)		57.2 (7.4)	56.3 (7.1)	57.5 (7.5)
Women (n, %)		571 (70.8%)	99 (44.8%)	472 (80.7%)*
BMI (SD)		32.1 (6.7)	25.9 (3.1)	34.5 (6.1)*
Waist (cm) (SD)		102.2 (14.8)	87.7 (8.6)	107.6 (12.9)*
Type 2 diabetes (n, %)^a^		315 (39.1%)	66 (29.9%)	249 (42.6%)*
Hypertension (n, %)		562 (69.7%)	132 (59.7%)	430 (73.5%)*
Smoking (n, %)	Non-smoker	376 (46.3%)	90 (40.7%)	283 (48.5%)
	Past-smoker	188 (23.2%)	57 (25.8%)	190 (32.5%)
	Current smoker	248 (30.5%)	75 (33.9%)	111 (19.0%)*
Alcohol use (n, %)	Non-drinker	229 (28.2%)	44 (19.9%)	182 (31.1%)
	Past-drinker	336 (41.4%)	71 (32.1%)	175 (30.0%)
	Current-drinker	246 (30.3%)	105 (47.5%)	228 (39.0%)*
Education		5.2 (2.8)	5.5 (2.8)	5.1 (2.8)
Physical activity score (SD)		31.4 (4.6)	32.6 (5.6)	31.1 (4.2)
Total energy intake (kcal, SD)		2167 (966)	2180 (859)	2162.4 (1003.9)

*Significant difference at T-test between the obese and non-obese group

^a^The number of participants with type 2 diabetes at the baseline

### Association between obesity and type 2 diabetes

To confirm the correlation between obesity and T2D, we first examined the association between obesity and T2D at bassline (n = 806). Both waist circumference (OR = 1.032, 95% CI 1.021–1.043, beta ± SE = 0.031 ± 0.006 cm, P = 1.20E-08) and BMI (OR = 1.031, 95% CI: 1.021–1.051, beta ± SE = 0.061 ± 0.012 kg/m^2^, P = 7.62E-06) were strongly associated with T2D prevalence at baseline when adjusting for sex, age, smoking, alcohol use, education and physical activity. To examine whether either BMI or waist were independently associated with T2D, we then controlled for BMI in the model, and found that waist circumference remained significantly associated with T2D (OR 1.031, 95% CI 1.012–1.051, beta ± SE = 0.031 ± 0.010 cm, P = 0.0015). On the other hand, when we controlled for waist circumference, BMI was no longer significantly associated with T2D (OR 1.003, 95% CI 0.961–1.047, beta  ± SE = 0.003 ± 0.022 kg/m^2^, P = 0.893).

Secondly, we also examined the association between obesity and T2D incidence at the 5-year follow-up. Among 806 participants, 412 were free of T2D at baseline. The general characteristics between the obese and non-obese groups at the baseline are shown in Table S1. Among them, 15.5% (n = 64) developed T2D at the 5-year follow-up, with 9.0% in the non-obese group and 19.2% in the obese group. Again, we found that waist circumference was highly associated with T2D incidence at 5-year follow-up, even after controlling for BMI (OR 1.053, 95% CI 1.031–1.075, beta  ± SE= 0.052 ± 0.017, P = 1.58E-06, and OR 1.06, 95% CI 1.06–1.022, beta  ± SE = 0.059 ± 0.019, P = 0.002). However, BMI was not associated with T2D incidence after controlling for waist circumference (OR 1.089, 95% CI 1.089–1.042, beta  ± SE = 0.085 ± 0.022, P = 0.0001, and OR 0.982, 95% CI 0.908–1.062, beta  ± SE = − 0.018 ± 0.040, P = 0.649). These observations suggest waist is a stronger predictor of T2D risk than BMI in this study population. Hence, we used waist circumference to define obesity for investigation of metabolomics links between obesity and metabolomics profiles.

### Plasma metabolites associating with type 2 diabetes

To identify metabolites that differ between T2D and non-T2D, we first examined the association between T2D and individual metabolites in all participants (n = 806) while adjusting for sex, age, education, physical activity, smoking, alcohol use, and total energy intake. There were 179 metabolites (Table S2) that were significantly associated with T2D prevalence after correcting for multiple testing based on FDR. Enrichment analysis based on Metabolon database identified six metabolic pathways that were overrepresented by the metabolites differing between T2D and non-T2D participants (Table S2). These include sphingomyelins (P = 4.69E-05), long chain fatty acids (P = 6.40E-03), phosphatidylinositol (P = 9.88E-03), glutamate metabolism (P = 0.0109), phosphatidylethanolamine (P = 0.019), and fatty acid metabolism (acyl choline) (P = 0.025).

Participants with a normal weight (non-obesity) can still develop T2D. Thus we identified the metabolites that showed significant differences between T2D and non-T2D status in participants (n = 221) with normal weight (non-obesity). We found 64 metabolites that are associated with T2D (Table S2). Six metabolic pathways were overrepresented by these metabolites, including sphingomyelin (P = 2.66E-06) common to those found in all participants (Table S2).

To identify metabolites that link obesity to T2D risk, we then examined the association between metabolites and T2D in participants (n = 585) who met the obesity criteria by waist (see section 2). After correction for multiple testing based on FDR and excluding those metabolites that were associated with T2D in participants without obesity, we found 120 metabolites that are significantly associated with T2D (Table S2). From enrichment analysis, six pathways were overrepresented by those metabolites. Among them, three pathways, long chain fatty acids (P = 7.48E-04), phosphatidylethanolamine (PE) (P = 7.96E-04), and fatty acid metabolism (acyl choline) (P = 2.39E-03) were shared with those found in all participants (Table 2).

**Table 2:** Metabolic pathways overrepresented by metabolites that are associated with type 2 diabetes

	Super pathway	Sub pathway	No. of metabolites assigned to the pathway	No. of significant metabolites in each pathway	Z score	P-value	FDR
All participants	Lipid	Sphingomyelins	18	15	4.229	2.35E-05	4.69E-05
(n = 806)	Lipid	Long chain fatty acid	12	9	2.820	4.80E-03	6.40E-03
	Lipid	Phosphatidylinositol (PI)	4	4	2.656	7.90E-03	9.88E-03
	Amino acid	Glutamate metabolism	9	7	2.609	9.09E-03	1.09E-02
	Lipid	Phosphatidylethanolamine (PE)	6	5	2.407	1.61E-02	1.88E-02
	Lipid	Fatty acid metabolism (acyl choline)	3	3	2.298	2.16E-02	2.47E-02
Non-obesity	Lipid	Sphingomyelins	18	9	4.84	1.30E-06	2.60E-06
(n = 221)	Carbohydrate	Fructose, mannose and galactose metabolism	4	3	3.75	1.77E-04	2.66E-04
	Lipid	Dihydrosphingomyelins	3	2	2.81	4.97E-03	6.62E-03
	Lipid	Plasmalogen	10	4	2.61	8.94E-03	1.12E-02
	Amino acid	Leucine, isoleucine and valine metabolism	20	6	2.37	1.78E-02	2.14E-02
	Lipid	Phosphatidylinositol (PI)	4	2	2.24	2.48E-02	2.90E-02
Obesity only	Lipid	Long chain fatty acid	12	8	3.56	3.74E-04	7.48E-04
(n = 585)	Lipid	Phosphatidylethanolamine (PE)	6	5	3.46	5.31E-04	7.96E-04
	Lipid	Fatty acid Metabolism (acyl choline)	3	3	3.12	1.79E-03	2.39E-03
	Cofactors and vitamins	Tocopherol metabolism	4	3	2.43	1.52E-02	1.90E-02
	Lipid	Polyunsaturated fatty acid (n3 and n6)	12	6	2.18	2.92E-02	3.51E-02

### Enrichment analysis—chemical classification

With these sets of statistically significantly different compounds, each unique to either the obese group (n = 120) or non-obese group (n = 12), we conducted in-depth enrichment analyses as these inform which biological functions are involved in T2D under different obesity conditions. These analyses were done in two different ways—functional categorization (eg., pathway and bioprocess enrichment) and chemical compound classification. This latter approach assesses enrichment of ontology or classification terms and is useful when identified metabolites are not assigned to pathways. The two- and three-tier hierarchical systems for classifying metabolites from Metabolon and LipidMaps, respectively were used, in addition to the classification system of Mbrole (Table S3). This latter tool identified 17 different fatty acids and conjugates within the obesity-T2D data, a significant enrichment for this molecular class (P = 1.09E-14). Members of this group include heptadecanoic acid, eicosadienoic acid, oleic acid, and docosapentaenoic acid (22n-3). We also noted a parallel if somewhat less robust enrichment in unsaturated fatty acids (P = 2.14E-07). Using LipidMaps classifications allowed us to identify a slight enrichment in acyl carnitines (P = 1.12E-04), whose members are octanoylcarnitine, tiglylcarnitine, 2-methylbutyrylcarnitine, and linoleoylcarnitine, which can serve as fuel for skeletal and cardiac muscle. Of note, the nonobese-T2D blood metabolite data had a slightly significant enrichment in phosphatidylinositols (P = 0.012) and a slight depletion in lipids (P = 0.022) (See Table S3).

### Enrichment analysis—pathways and bioprocesses

Differences in metabolite levels between T2D and non-T2D within and unique to the obese group indicated that several pathways were enriched, including biosynthesis of unsaturated fatty acids (P = 3.96E-07), carnitine synthesis (P = 1.22E-02), phospholipid/phosphatidylcholine to linoleate conversion (P = 1.35E-02), glutamate biosynthesis (P = 1.22E-02), and phosphatidylcholine biosynthesis (P = 3.92E-02). Similarly, for the metabolites unique to the non-obese group, different pathways were noted as enriched, but these were small in number, likely owing to the reduced number of significantly different metabolites in this analysis group. We note slight enrichments in glyoxylate/dicarboxylate metabolism and nicotinate/nicotinamide metabolism, driven by tartarate and maleate, respectively (both with P = 0.049).

The malate-aspartate shuttle produces glutamate upon glucose stimulation, which supports the stimulatory effect of incretins and glutamate uptake into insulin granules, thereby promoting insulin secretion (Gheni et al., 2014).We confirmed the associations between plasma metabolites and T2D incidence at 5-year follow-up.

As we showed obesity was strongly associated with T2D not only at the baseline, but also T2D incidence at 5-year follow-up, we tested whether the 120 metabolites that were associated with T2D in obese participants at base line were associated with future T2D incidence (i.e., 5-year follow-up). We conducted the test only in those participants free of T2D at baseline (n = 412). Among the 120 metabolites, 24 were associated with T2D incidence at 5-year follow-up (Table S4). Based on the enrichment analysis, three metabolic pathways were enriched: phosphatidylethanolamine (P = 2.24E-17), long chain fatty acid (P = 4.34E-12), and glutamate metabolism (P = 3.02E-02) (Table 3). Both phosphatidylethanolamine and long chain fatty acid were overrepresented among all participants and participants with obesity only. Strikingly, all these 13 metabolites in three pathways were positively correlated with T2D at the baseline and T2D incidence at the 5-year follow-up (Table 4). This observation suggests that three over-representative pathways are “molecular mechanisms” linking obesity to T2D risk.

**Table 3:** Metabolic pathways overrepresented by the metabolites unique to obesity and T2D, that are associated with type 2 diabetes incidence at the 5-year follow-up

Super pathway	Sub pathway	No. of metabolites assigned to the pathway	No. of significant metabolites in each pathway	Z score	P-value	FDR
Lipid	Phosphatidylethanolamine (PE)	6	5	8.561	1.12E-17	2.24E-17
Lipid	Long chain fatty acid	12	6	6.983	2.89E-12	4.34E-12
Amino acid	Glutamate metabolism	9	2	2.279	2.27E-02	3.02E-02

**Table 4:** Thirteen metabolites unique to type 2 diabetes and abdominal obesity associated with type 2 incidence at 5-year follow-up

Chemical ID	Biochemical	Sub pathway	T2D in participants with obesity			T2D incidence at 5-year follow-up		
			Beta	SE	P-value	Beta	SE	P-Value
561	Glutamate	Glutamate metabolism	2.191	0.674	8.54E-04	2.705	0.933	3.96E-03
100002679	Gamma-carboxyglutamate	Glutamate metabolism	0.564	0.232	1.07E-02	1.137	0.44	7.96E-03
519	Myristate (14:0)	Long chain fatty acid	0.602	0.212	4.08E-03	1.036	0.37	5.47E-03
424	Palmitate (16:0)	Long chain fatty acid	1.432	0.335	1.07E-05	1.717	0.561	2.04E-03
891	Margarate (17:0)	Long chain fatty acid	1.435	0.293	2.09E-07	1.386	0.487	4.54E-03
100001278	10-heptadecenoate (17:1n7)	Long chain fatty acid	0.586	0.162	2.24E-04	0.625	0.283	2.94E-02
439	Stearate (18:0)	Long chain fatty acid	2.321	0.524	4.08E-06	2.437	0.818	2.89E-03
100001277	10-nonadecenoate (19:1n9)	Long chain fatty acid	0.813	0.184	3.35E-06	0.709	0.304	2.05E-02
1526	1-palmitoyl-2-oleoyl-GPE (16:0/18:1)	Phosphatidylethanolamine (PE)	0.356	0.078	8.90E-07	0.285	0.099	5.52E-03
100001870	1-palmitoyl-2-linoleoyl-GPE (16:0/18:2)	Phosphatidylethanolamine (PE)	0.257	0.081	1.07E-03	0.365	0.135	8.10E-03
100008990	1-palmitoyl-2-arachidonoyl-GPE (16:0/20:4)	Phosphatidylethanolamine (PE)	0.642	0.121	1.64E-08	0.507	0.172	3.89E-03
100008976	1-stearoyl-2-linoleoyl-GPE (18:0/18:2)	Phosphatidylethanolamine (PE)	0.253	0.091	4.42E-03	0.347	0.144	1.78E-02
100008977	1-stearoyl-2-arachidonoyl-GPE (18:0/20:4)	Phosphatidylethanolamine (PE)	0.653	0.134	3.90E-07	0.529	0.207	1.15E-02

### Food groups associated with T2D-associated metabolites unique to obesity

Given that 13 metabolites identified as T2D-biomarkers and unique to obesity were assigned to three metabolic pathways, we then investigated what types of food were associated with these metabolites. Using linear regression models, we identified the top food group associated with each metabolite while adjusted for age, sex, alcohol use, smoking, education, physical activity, and total energy intake (Table 5). Two metabolites (glutamate and gamma-carboxyglutamate) in glutamate metabolism and three of five metabolites (1-palmitoyl-2-oleoyl-GPE, 1-palmitoyl-2-linoleoyl-GPE, and 1-stearoyl-2-linoleoyl-GPE) in phosphatidylethanolamine (PE) were highly associated with soft drink intake (servings). Three of six metabolites (margarate, 10-heptadecenoate, and 10-nonadecenoate) in the long chain fatty acid pathway were positively associated with energy-dense takeout food, like pizza (servings). Interestingly, in the same pathway, two metabolites (palmitate and stearate) were correlated with candy/sugar intake (servings).

**Table 5:** Thirteen metabolites unique to T2D and abdominal obesity associated with dietary food groups

CompID	Chemical ID	Biochemical^a^	Sub pathway	Food group^b^	Beta	SE	P-value*
57	561	Glutamate	Glutamate metabolism	servings_SSB	0.054	0.013	3.55E-05
38754	100002679	Gamma-carboxyglutamate	Glutamate metabolism	servings_SSB	0.028	0.011	8.00E-03
1365	519	Myristate (14:0)	Long chain fatty acid	servings_Starchy vegetable	− 0.092	0.044	3.76E-02
1336	424	Palmitate (16:0)	Long chain fatty acid	servings_candy/sugar	− 0.015	0.007	2.60E-02
1121	891	Margarate (17:0)	Long chain fatty acid	servings_Pizza	0.129	0.065	4.73E-02
33971	100001278	10-heptadecenoate (17:1n7)	Long chain fatty acid	servings_Pizza	0.186	0.090	3.80E-02
1358	439	stearate (18:0)	Long Chain Fatty Acid	servings_candy/sugar	− 0.010	0.004	1.61E-02
33972	100001277	10-nonadecenoate (19:1n9)	Long Chain Fatty Acid	servings_Pizza	0.205	0.092	2.61E-02
19263	1526	1-palmitoyl-2-oleoyl-GPE (16:0/18:1)	Phosphatidylethanolamine (PE)	servings_SSB	0.058	0.020	3.87E-03
42449	100001870	1-palmitoyl-2-linoleoyl-GPE (16:0/18:2)	Phosphatidylethanolamine (PE)	servings_SSB	0.055	0.019	3.28E-03
52464	100008990	1-palmitoyl-2-arachidonoyl-GPE (16:0/20:4)	Phosphatidylethanolamine (PE)	servings_Soup	0.151	0.059	1.03E-02
52,446	100008976	1-stearoyl-2-linoleoyl-GPE (18:0/18:2)	Phosphatidylethanolamine (PE)	servings_SSB	0.050	0.017	3.36E-03
52447	100008977	1-stearoyl-2-arachidonoyl-GPE (18:0/20:4)	Phosphatidylethanolamine (PE)	servings_Fish	0.126	0.061	3.96E-02

*All beta and P values were calculated based on a linear regression model adjusted for age, sex, alcohol use, smoking, education, physical activity, and total energy intake. *SSB* sugar sweeten beverages

^a^Metabolite concentrations were natural log-transformed

^b^Food groups were defined in the methods; CompID is the compound identifier (Metabolon)

## Discussion

This study confirms the strong association between abdominal obesity and increased risk of T2D at baseline and incidence of T2D at 5-year follow-up among this unique population of Puerto Ricans living in the US. We identified a set of T2D-associated metabolites unique to obese individuals in this population. Enrichment analysis identified key metabolic pathways that were highly overrepresented by such metabolites. Similar enrichment was confirmed among metabolites that differed between T2D incidence and non-T2D at the 5-year follow-up. Furthermore, among 24 metabolites that were confirmed in the 5-year T2D incidence, we identified 13 metabolites that were correlated with sugar-sweetened beverage (SSB, energy-dense takeout food (like pizza), and sugar intake, two of which are known risk dietary factors for T2D. These observations suggest that energy-dense takeout food and SSB consumption are associated with three metabolic pathways that appear to contribute to T2D development among obese individuals.

There is overwhelming evidence that obesity is a causal factor of T2D (Kodama et al., 2014). Consistent with non-Hispanic populations (CDC, 2012), over 90% of participants with T2D in this population are overweight or obese. In this study, our results showed that abdominal obesity (defined by waist circumference) is a stronger indicator of T2D risk than BMI, suggesting that fat deposition is the key risk factor for T2D in obesity (Smith, 2015). However, not all obese participants developed T2D. Over the 5-year follow-up, 19% of obese participants developed T2D, whereas only 7.1% of participants with normal weight did. This gives rise to the notion that there are distinct mechanisms underlying the development of obese-associated T2D and T2D occurring in the non-obese.

A substantial number of observational and intervention studies identified metabolic markers that differ between T2D and non-T2D conditions (Ahola-Olli et al., 2019; Guasch-Ferre et al., 2016). Most metabolomics biomarkers were described in the populations of European and Asian ancestries. Our findings shared some common metabolites and pathways with other populations, such as branched chain amino acids (BCAA), phospholipids, triacylglycerols, ketone bodies, sphingomyelins, acyl-carnitines and organic acids (Fall et al., 2016; Liu et al., 2017; Mahendran et al., 2013; Tillin et al., 2015; Wurtz et al., 2013).

We also noted metabolites and pathways unique to this Hispanic population, including unsaturated fatty acids and hydroxyl fatty acids. The category of unsaturated fatty acids [FA0103 in LipidMaps] has 1.16-fold enrichment in all (n = 172) abdominal obesity-associated metabolites but is not significant (P = 0.27). However, in the subset of metabolites unique to abdominal obesity (n = 120), the fold enrichment is 1.47 and reaches significance (P = 0.017). In T2D, unsaturated fatty acids are elevated, impairing ABCA1 efflux of cholesterol via phospholipase D2 stimulation (Wang & Oram, 2005).

The hydroxy fatty acids category [FA0105 in LipidMaps] shows significant enrichment in both obese and normal weight participants. This significance is lost when examining the set of metabolites unique to obese-waist, indicating that the connections between hydroxy fatty acids and diabetes are independent of obesity/adiposity. 3-hydroxyhexanoic acid is found in serum and urine of diabetics with ketoacidosis (Niwa et al., 1985). HACR3 is a receptor for the beta-oxidation intermediate 3-OH-octanoic acid, which has antilipolytic activity on human adipocytes. HACR3 is coupled to G_i_-type G-proteins and is activated by 2- and 3-OH-octanoic acid with EC50 values of about 4 and 8 microM, respectively. Interestingly, 3-OH-octanoic acid plasma concentrations reach micromolar concentrations under conditions of increased beta-oxidation rates, like in diabetic ketoacidosis or under a ketogenic diet. These data suggest that the ligand receptor pair 3-OH-octanoic acid/HACR3 mediates in humans a negative feedback regulation of adipocyte lipolysis to counteract prolipolytic influences under conditions of physiological or pathological increases in beta-oxidation rates (Ahmed et al., 2009). 3-OH-isobutyrate is a valine byproduct, and with FGF21 might be involved in protein-mediated insulin resistance in humans (Harris et al., 2017). Total beta-hydroxy fatty acid (BHFA) content increases progressively with age in diabetic hearts in mice. Oxidative phosphorylation studies using isolated mitochondria from diabetic mice demonstrated depressed state 3 oxidation rates with both palmityl carnitine and pyruvate as substrates. There was a significant decrease in mitochondrial total NAD + NADH content in diabetic hearts. The data indicate that deficiencies in total NAD + and NADH content can account for the depressed state 3 oxidation of palmitylcarnitine and pyruvate in diabetic mice that in turn may explain the abnormal accumulation of BFHA (Kuo et al., 1983). Carnitine synthesis is one pathway noted in our analyses as significantly enriched in the obese-T2D group.

Low sphingomyelin could arise from higher aSMase (SMPD1) activity, and high levels of aSMase have been correlated with insulin resistance and diabetes (Deevska et al., 2009). Downregulation of SMS2 (SGMS2) activity results in protective effects against obesity, atherosclerosis and diabetes and makes SMS2 inhibitors potential medicines (Chen & Cao, 2017). Lower serum sphingomyelins associated with MetS, lower HDL and lower TG, but not with glucose in two cohorts. No relationship to blood pressure and waist circumference were reported (Mahajan et al., 2020). Interestingly, we note a slightly significant enrichment for the endothelial nitric oxide synthase (NOS3) pathway (P = 0.026) in the obesity-T2D data, which may relate to impaired peripheral vascular integrity.

Obesity increases the risk of T2D, and this increased risk may be attributed to adipose dysfunction, leading to the release of cytokines that inhibit insulin signaling and subsequent release of NEFA and glycerol (Smith, 2015). This study identified three pathways: phosphatidylethanolamine (PE), long-chain fatty acids, and glutamate metabolism, which were represented by 13 metabolites (Table 4), and supplemental analysis by other metabolomics tools also highlighted phosphatidylcholine biosynthesis. In particular, we identified four phosphatidylethanolamine (PE) and six long chain fatty acids. PC and PE content of skeletal muscle responds positively to exercise, and PC:PE ratio is inversely related to insulin sensitivity (Lee et al., 2018). Obesity-related T2D is attributed partially to energy substrate imbalance both in the circulation and more importantly in the major effector organs of the diabetic condition: liver, skeletal muscle, and pancreatic beta-cell islets. Exercise enhances insulin sensitivity, skeletal muscle PC by 21% and PE by 42%, reduces PC:PE ratio by16%. PC:PE ratio correlated negatively with insulin sensitivity (Lee et al., 2018). Increased long chain fatty acids and PE observed in this study reflected the high risk of T2D in obese participants. More strikingly, four PE and long chain fatty acids were positively correlated with SSB intake and pizza consumption in this population.

Plasma levels of glycosphingolipids, including six sphingomyelins and two dihydrosphingomyelins, are lower in T2D (beta < 0, Table S2). These sphingolipids carry a double bond, and the presence of a double bond in ceramide (sphingolipid) can promote lipid uptake and storage, and impair glucose utilization (Chaurasia et al., 2019). Decreases of several long and very long chain lactosylceramides were significantly associated with increased risk of macroalbuminuria but not chronic kidney disease in T1D (Lopes-Virella et al., 2019). In Hispanic Community Health Study/Study of Latinos, glycosylceramides, lactosylceramides, or other unsaturated sphingomyelins (even if having a saturated fatty acid base) were not associated with risk of diabetes; positive associations observed with a ceramide score and a score of sphingomyelins with fully saturated sphingoid-fatty acid pairs were attenuated after adjusting for triglyceride (TG) (Chen et al., 2020). Lower lactosylceramide with palmitic acid (LC-16) associated with higher glucose levels at baseline in Strong Heart Family, and may indicate impaired glucose metabolism (Jensen et al., 2019).

A noted strength of this study is the high prevalence of both central obesity and T2D and a large number of metabolites examined, giving the analyses herein a statistical power. In addition to baseline results, this study provided data on T2D incidence at the 5-year follow-up, which provided the opportunity to examine both cross-sectional and longitudinal associations between metabolites and T2D. On the other hand, this study has limitations in that the metabolites were measured only in plasma samples collected at baseline. Replication in other Hispanic populations and randomized controlled trials are needed in future studies.

In summary, in this Hispanic population with a high prevalence of obesity and T2D, we identified T2D-associated metabolites and metabolic pathways unique to abdominal obesity. Thirteen metabolites, over-represented in three metabolic pathways, were correlated with three food groups, linking dietary behavior to obesity and T2D risk. Our findings provide strong evidence supporting the role of abdominal obesity in contributing to T2D, and improve understanding of the mechanisms underlying the development of T2D, facilitating the development of strategies for the prevention of T2D in Hispanic populations.

## Supplementary Information

Below is the link to the electronic supplementary material.

Supplementary file1 (DOCX 112 kb)

## Abbreviations

ABCA1: ATP binding cassette subfamily A member 1
BHFA: Beta-hydroxy fatty acid
BMI: Body mass index
BPRHS: Boston Puerto Rican Health Study
FDR: False discovery rate
FFQ: Food frequency questionnaire
FPG: Fasting plasma glucose
OR: Odds ratio
PC: Phosphatidylcholine
PE: Phosphatidylethanolamine
SSB: Sugar-sweetened beverages
T2D: Type 2 diabetes
TG: Triglyceride

## References

[CR1] Abdullah A, Stoelwinder J, Shortreed S, Wolfe R, Stevenson C, Walls H, De Courten M, Peeters A (2011). The duration of obesity and the risk of type 2 diabetes. Public Health Nutrition.

[CR2] Ahmed K, Tunaru S, Langhans CD, Hanson J, Michalski CW, Kolker S, Jones PM, Okun JG, Offermanns S (2009). Deorphanization of GPR109B as a receptor for the beta-oxidation intermediate 3-OH-octanoic acid and its role in the regulation of lipolysis. Journal of Biological Chemistry.

[CR3] Ahola-Olli AV, Mustelin L, Kalimeri M, Kettunen J, Jokelainen J, Auvinen J, Puukka K, Havulinna AS, Lehtimaki T, Kahonen M, Juonala M, Keinanen-Kiukaanniemi S, Salomaa V, Perola M, Jarvelin MR, Ala-Korpela M, Raitakari O, Wurtz P (2019). Circulating metabolites and the risk of type 2 diabetes: A prospective study of 11,896 young adults from four finnish cohorts. Diabetologia.

[CR4] Benjamini YA, Yosef H (1995). Controlling the false discovery rate: A practical and powerful approach to multiple testing. Journal of the Royal Statistical Society: Series B (Methodological).

[CR5] Boden G (1998). Free fatty acids (FFA), a link between obesity and insulin resistance. Frontiers in Bioscience.

[CR6] CDC (2012). Diabetes report card 2012.

[CR7] Chaurasia B, Tippetts TS, Mayoral Monibas R, Liu J, Li Y, Wang L, Wilkerson JL, Sweeney CR, Pereira RF, Sumida DH, Maschek JA, Cox JE, Kaddai V, Lancaster GI, Siddique MM, Poss A, Pearson M, Satapati S, Zhou H, Summers SA (2019). Targeting a ceramide double bond improves insulin resistance and hepatic steatosis. Science.

[CR8] Chen GC, Chai JC, Yu B, Michelotti GA, Grove ML, Fretts AM, Daviglus ML, Garcia-Bedoya OL, Thyagarajan B, Schneiderman N, Cai J, Kaplan RC, Boerwinkle E, Qi Q (2020). Serum sphingolipids and incident diabetes in a US population with high diabetes burden: The Hispanic Community Health Study/Study of Latinos (HCHS/SOL). American Journal of Clinical Nutrition.

[CR9] Chen Y, Cao Y (2017). The sphingomyelin synthase family: proteins, diseases, and inhibitors. Biological Chemistry.

[CR10] Deevska GM, Rozenova KA, Giltiay NV, Chambers MA, White J, Boyanovsky BB, Wei J, Daugherty A, Smart EJ, Reid MB, Merrill AH, Nikolova-Karakashian M (2009). Acid sphingomyelinase deficiency prevents diet-induced hepatic triacylglycerol accumulation and hyperglycemia in mice. Journal of Biological Chemistry.

[CR100] Evans AM, DeHaven CD, Barrett T, Mitchell M, Milgram E (2009). Integrated, nontargeted ultrahigh performance liquidchromatography/electrospray ionization tandem mass spectrometry platform for the identification and relativequantification of the small-molecule complement of biological systems. Analytical Chemistry.

[CR11] Fabregat A, Jupe S, Matthews L, Sidiropoulos K, Gillespie M, Garapati P, Haw R, Jassal B, Korninger F, May B, Milacic M, Roca CD, Rothfels K, Sevilla C, Shamovsky V, Shorser S, Varusai T, Viteri G, Weiser J, D’eustachio P (2018). The reactome pathway knowledgebase. Nucleic Acids Research.

[CR12] Fall T, Salihovic S, Brandmaier S, Nowak C, Ganna A, Gustafsson S, Broeckling CD, Prenni JE, Kastenmuller G, Peters A, Magnusson PK, Wang-Sattler R, Giedraitis V, Berne C, Gieger C, Pedersen NL, Ingelsson E, Lind L (2016). Non-targeted metabolomics combined with genetic analyses identifies bile acid synthesis and phospholipid metabolism as being associated with incident type 2 diabetes. Diabetologia.

[CR13] Gheni G, Ogura M, Iwasaki M, Yokoi N, Minami K, Nakayama Y, Harada K, Hastoy B, Wu X, Takahashi H, Kimura K, Matsubara T, Hoshikawa R, Hatano N, Sugawara K, Shibasaki T, Inagaki N, Bamba T, Mizoguchi A, Seino S (2014). Glutamate acts as a key signal linking glucose metabolism to incretin/cAMP action to amplify insulin secretion. Cell Reports.

[CR14] Guasch-Ferre M, Hruby A, Toledo E, Clish CB, Martinez-Gonzalez MA, Salas-Salvado J, Hu FB (2016). Metabolomics in prediabetes and diabetes: A systematic review and meta-analysis. Diabetes Care.

[CR15] Han M (2020). The dose-response relationship between alcohol consumption and the risk of type 2 diabetes among Asian men: A systematic review and meta-analysis of prospective cohort studies. Journal of Diabetes Research.

[CR16] Harris LLS, Smith GI, Patterson BW, Ramaswamy RS, Okunade AL, Kelly SC, Porter LC, Klein S, Yoshino J, Mittendorfer B (2017). Alterations in 3-hydroxyisobutyrate and FGF21 metabolism are associated with protein ingestion-induced insulin resistance. Diabetes.

[CR17] Hirst JA, Aronson JK, Feakins BG, Ma C, Farmer AJ, Stevens RJ (2017). Short- and medium-term effects of light to moderate alcohol intake on glycaemic control in diabetes mellitus: A systematic review and meta-analysis of randomized trials. Diabetic Medicine.

[CR18] Jensen PN, Fretts AM, Yu C, Hoofnagle AN, Umans JG, Howard BV, Sitlani CM, Siscovick DS, King IB, Sotoodehnia N, Mcknight B, Lemaitre RN (2019). Circulating sphingolipids, fasting glucose, and impaired fasting glucose: The strong heart family study. EBioMedicine.

[CR19] Kodama S, Horikawa C, Fujihara K, Yoshizawa S, Yachi Y, Tanaka S, Ohara N, Matsunaga S, Yamada T, Hanyu O, Sone H (2014). Quantitative relationship between body weight gain in adulthood and incident type 2 diabetes: A meta-analysis. Obesity Reviews.

[CR20] Kuo TH, Moore KH, Giacomelli F, Wiener J (1983). Defective oxidative metabolism of heart mitochondria from genetically diabetic mice. Diabetes.

[CR101] Lee, S., Norheim, F., Gulseth, H. L., Langleite, T. M., Aker, A., Gundersen, T. E., Holen, T., Birkeland, K. I. & Drevon, C. A. (2018). Skeletal muscle phosphatidylcholine and phosphatidylethanolamine respond to exercise and influence insulin sensitivity in men. *Scientific Reports, 8*(1), 6531. 10.1038/s41598-018-24976-x. Erratum in: Scientific Reports. 2018;8(1):7885. 10.1038/s41598-018-24976-x PMC5916947 29695812

[CR21] Liu JJ, Ghosh S, Kovalik JP, Ching J, Choi HW, Tavintharan S, Ong CN, Sum CF, Summers SA, Tai ES, Lim SC (2017). Profiling of plasma metabolites suggests altered mitochondrial fuel usage and remodeling of sphingolipid metabolism in individuals with type 2 diabetes and kidney disease. Kidney International Reports.

[CR22] Lopes-Virella MF, Baker NL, Hunt KJ, Hammad SM, Arthur J, Virella G, Klein RL, DER Group (2019). Glycosylated sphingolipids and progression to kidney dysfunction in type 1 diabetes. Journal of Clinical Lipidology.

[CR23] Lopez-Ibanez J, Pazos F, Chagoyen M (2016). Mbrole 2.0-functional enrichment of chemical compounds. Nucleic Acids Research.

[CR24] Magkos F, Hjorth MF, Astrup A (2020). Diet and exercise in the prevention and treatment of type 2 diabetes mellitus. Nature Reviews Endocrinology.

[CR25] Mahajan UV, Varma VR, Huang CW, An Y, Tanaka T, Ferrucci L, Takebayashi T, Harada S, Iida M, Legido-Quigley C, Thambisetty M (2020). Blood metabolite signatures of metabolic syndrome in two cross-cultural older adult cohorts. International Journal of Molecular Sciences.

[CR26] Mahendran Y, Vangipurapu J, Cederberg H, Stancakova A, Pihlajamaki J, Soininen P, Kangas AJ, Paananen J, Civelek M, Saleem NK, Pajukanta P, Lusis AJ, Bonnycastle LL, Morken MA, Collins FS, Mohlke KL, Boehnke M, Ala-Korpela M, Kuusisto J, Laakso M (2013). Association of ketone body levels with hyperglycemia and type 2 diabetes in 9398 Finnish men. Diabetes.

[CR27] Malik VS, Popkin BM, Bray GA, Despres JP, Willett WC, Hu FB (2010). Sugar-sweetened beverages and risk of metabolic syndrome and type 2 diabetes: A meta-analysis. Diabetes Care.

[CR28] Mangano KM, Noel SE, Lai CQ, Christensen JJ, Ordovas JM, Dawson-Hughes B, Tucker KL, Parnell LD (2021). Diet-derived fruit and vegetable metabolites show sex-specific inverse relationships to osteoporosis status. Bone.

[CR29] Mitri J, Mohd Yusof BN, Maryniuk M, Schrager C, Hamdy O, Salsberg V (2019). Dairy intake and type 2 diabetes risk factors: A narrative review. Diabetes and Metabolic Syndrome: Clinical Research and Reviews.

[CR30] Niwa T, Yamada K, Ohki T, Furukawa H (1985). 3-Hydroxyhexanoic acid: An abnormal metabolite in urine and serum of diabetic ketoacidotic patients. Journal of Chromatography.

[CR31] Noel SE, Newby PK, Ordovas JM, Tucker KL (2009). A traditional rice and beans pattern is associated with metabolic syndrome in Puerto Rican older adults. Journal of Nutrition.

[CR32] Paffenbarger RS, Hyde RT, Wing AL, Lee IM, Jung DL, Kampert JB (1993). The association of changes in physical-activity level and other lifestyle characteristics with mortality among men. New England Journal of Medicine.

[CR33] Rice Bradley BH (2018). Dietary fat and risk for type 2 diabetes: A review of recent research. Curr Nutr Rep.

[CR34] Roden M, Shulman GI (2019). The integrative biology of type 2 diabetes. Nature.

[CR35] Sainsbury E, Kizirian NV, Partridge SR, Gill T, Colagiuri S, Gibson AA (2018). Effect of dietary carbohydrate restriction on glycemic control in adults with diabetes: A systematic review and meta-analysis. Diabetes Research and Clinical Practice.

[CR36] Serrano Rios M (1998). Relationship between obesity and the increased risk of major complications in non-insulin-dependent diabetes mellitus. European Journal of Clinical Investigation.

[CR37] Smith U (2015). Abdominal obesity: A marker of ectopic fat accumulation. The Journal of Clinical Investigation.

[CR38] Storey JD (2002). A direct approach to false discovery rates. Journal of the Royal Statistical Society Series B (Statistical Methodology).

[CR39] Sumner LW, Amberg A, Barrett D, Beale MH, Beger R, Daykin CA, Fan TW, Fiehn O, Goodacre R, Griffin JL, Hankemeier T, Hardy N, Harnly J, Higashi R, Kopka J, Lane AN, Lindon JC, Marriott P, Nicholls AW, Viant MR (2007). Proposed minimum reporting standards for chemical analysis chemical analysis working group (CAWG) metabolomics standards initiative (MSI). Metabolomics.

[CR40] Tillin T, Hughes AD, Wang Q, Wurtz P, Ala-Korpela M, Sattar N, Forouhi NG, Godsland IF, Eastwood SV, Mckeigue PM, Chaturvedi N (2015). Diabetes risk and amino acid profiles: Cross-sectional and prospective analyses of ethnicity, amino acids and diabetes in a South Asian and European cohort from The Sabre (Southall and Brent revisited) study. Diabetologia.

[CR41] Toi PL, Anothaisintawee T, Chaikledkaew U, Briones JR, Reutrakul S, Thakkinstian A (2020). Preventive role of diet interventions and dietary factors in type 2 diabetes mellitus: An umbrella review. Nutrients.

[CR42] Tucker KL, Bianchi LA, Maras J, Bermudez OI (1998). Adaptation of a food frequency questionnaire to assess diets of Puerto Rican and non-Hispanic adults. American Journal of Epidemiology.

[CR43] Tucker KL, Mattei J, Noel SE, Collado BM, Mendez J, Nelson J, Griffith J, Ordovas JM, Falcon LM (2010). The Boston Puerto Rican Health Study, a longitudinal cohort study on health disparities In Puerto Rican adults: Challenges and opportunities. BMC Public Health.

[CR44] Wang Y, Oram JF (2005). Unsaturated fatty acids phosphorylate and destabilize ABCA1 through a phospholipase D2 pathway. Journal of Biological Chemistry.

[CR45] Wulff JE, Mitchell M (2018). A comparison of various normalization methods for LC/MS metabolomics data. Advances in Bioscience and Biotechnology.

[CR46] Wurtz P, Soininen P, Kangas AJ, Ronnemaa T, Lehtimaki T, Kahonen M, Viikari JS, Raitakari OT, Ala-Korpela M (2013). Branched-chain and aromatic amino acids are predictors of insulin resistance in young adults. Diabetes Care.

[CR47] Zhao LG, Zhang QL, Liu XL, Wu H, Zheng JL, Xiang YB (2019). Dietary protein intake and risk of type 2 diabetes: A dose-response meta-analysis of prospective studies. European Journal of Nutrition.

[CR48] Zhou B, Ichikawa R, Parnell LD, Noel SE, Zhang X, Bhupathiraju SN, Smith CE, Tucker KL, Ordovas JM, Lai CQ (2020). Metabolomic links between sugar-sweetened beverage intake and obesity. Journal of Obesity.

