# Supplementary material for: Metabolite patterns link diet, obesity, and type 2 diabetes in a Hispanic population
Source: Metabolomics. 2021 Sep 22;17(10):88. doi: 10.1007/s11306-021-01835-x (PMC8458177; doi:10.1007/s11306-021-01835-x)

**Metabolite patterns link diet, obesity, and type 2 diabetes in a Hispanic population**

Laurence D. Parnell, Sabrina E. Noel, Shilpa N. Bhupathiraju, Caren E. Smith, Danielle E. Haslam, Xiyuang Zhang, Katherine L. Tucker, Jose M. Ordovas, Chao-Qiang Lai

**Supplementary materials**

| **Supplemental Table 1. General characteristics of participants without T2D at the baseline and at 5-year follow-up in BPRHS** | | | | | | | | |
| --- | --- | --- | --- | --- | --- | --- | --- | --- |
|  |  | Baseline | | |  | 5-year follow-up | | |
|  |  | Total (n=412) | Non-obese (n=126) | Obese (n=286) |  | Total (n=412) | Non-obese (n=126) | Obese (n=286) |
| Age (SD) |  | 56.2 (7.6) | 55.1 (7.0) | 56.6 (7.8) |  | 62.3 (7.8) | 61.3 (7.1) | 62.8 (8.0) |
| Women (n,%) |  | 306 (74.3%) | 62 (49.2%) | 244 (85.3%)* | | 306 (74.3%) | 62 (49.2) | 244 (85.3%) |
| BMI (SD) |  | 31.3 (6.5) | 25.6 (3.4) | 33.9 (6.0)* |  | 30.4 (6.2) | 25.9 (4.3) | 32.5 (5.8) |
| Waist (cm) (SD) |  | 99.5 (14.0) | 86.6 (9.0) | 105.2 (12.0)* | | 102.3 (17.2) | 94.0 (13.3) | 106.2 (17.5) |
| Fasting glucose(mg/dl) (SD) | | 97.0 (10.5) | 94.3 (9.6) | 98.3 (10.7) |  | 97.6 (21.1) | 96.0 (25.6) | 98.3 (18.8) |
| ^#^Type 2 diabetes incidence |  | 0 | 0 | 0 |  | 64 (15.5%) | 9 (7.1%) | 55 (19.2%)* |
| Hypertension (n, %) |  | 256 (62.1%) | 67 (53.2%) | 189 (66.1%)* | | 256 (62.1%) | 69 (54.7%) | 187 (65.4%) |
| Smoking (n,%) | Non-smoker | 196 (47.7%) | 47 (37.3%) | 149 (52.3%) |  | 190 (46.7%) | 46 (36.8%) | 144 (51.1%) |
|  | Past-smoker | 101 (24.6%) | 32 (25.4%) | 82 (28.8%) |  | 78 (19.2%) | 36 (28.8%) | 42 (14.9%) |
|  | Current smoker | 114 (27.7%) | 47 (37.3%) | 54 (19.0%)* |  | 139 (34.2%) | 43 (34.4%) | 96 (34.0%) |
| Alcohol use (n, %) | Non-drinker | 116 (28.35) | 28 (22.4%) | 88 (30.9%) |  | 289 (72.8%) | 80 (65.0%) | 209 (76.3%) |
|  | Past-drinker | 187 (45.6%) | 33 (26.4%) | 74 (26.0%) |  |  |  |  |
|  | Current-drinker | 107 (26.1%) | 64 (51.2%) | 123 (43.2%) |  | 108 (27.2%) | 43 (35.0%) | 65 (23.7%) |
| Education |  | 5.3 (2.8) | 5.6 (2.8) | 5.1 (2.8) |  |  |  |  |
| Physical activity score (SD) |  | 31.7 (4.6) | 32.9 (5.6) | 31.2 (4.0) |  | 31.8 (6.2) | 32.3 (6.6) | 31.5 (5.9) |
| Total energy intake (kcal, SD) | | 2174 (879) | 2185 (718) | 2169 (942) |  | 1645 (884) | 1854 (1033) | 1551 (794) |
| ^#^The number of type 2 diabetes incidence at the 5-year follow-up. All participants were normal at the baseline. | | | | | | | |  |

*Significant difference at T-test between the obese and non-obese group.

| **Supplemental Table 2. Metabolites were associated with T2D in three groups of BPRHS participants (n=805, n=221, n=586)** | | | | | |  |  |
| --- | --- | --- | --- | --- | --- | --- | --- |
|  | CompID | BIOCHEMICAL | P-value | FDR | | Beta | SE |
| All participants | 20675 | 1,5-anhydroglucitol (1,5-AG) | 2.95E-57 | | 1.55E-54 | -3.237 | 0.248 |
| (n=805) | 48153 | mannose | 6.84E-54 | | 1.80E-51 | 3.314 | 0.283 |
|  | 587 | gluconate | 6.01E-29 | | 1.05E-26 | 1.104 | 0.122 |
|  | 53242 | 5-bromotryptophan | 4.02E-24 | | 5.28E-22 | -1.819 | 0.197 |
|  | 27731 | ribonate | 2.06E-22 | | 1.80E-20 | 1.599 | 0.191 |
|  | 48195 | fructose | 1.86E-22 | | 1.95E-20 | 1.025 | 0.123 |
|  | 52281 | 2-hydroxybutyrate/2-hydroxyisobutyrate | 5.04E-20 | | 3.79E-18 | 1.215 | 0.147 |
|  | 52984 | 3-hydroxybutyrylcarnitine (2) | 7.04E-19 | | 4.63E-17 | 1.187 | 0.158 |
|  | 1549 | 3-hydroxyisobutyrate | 1.93E-16 | | 1.13E-14 | 1.194 | 0.166 |
|  | 22001 | 3-hydroxyoctanoate | 1.43E-15 | | 7.51E-14 | 0.984 | 0.147 |
|  | 22053 | 3-hydroxydecanoate | 1.57E-15 | | 7.51E-14 | 1.064 | 0.149 |
|  | 1303 | malate | 3.33E-15 | | 1.46E-13 | 3.014 | 0.436 |
|  | 60 | leucine | 6.51E-15 | | 2.45E-13 | 2.809 | 0.390 |
|  | 52437 | sphingomyelin (d18:2/24:1, d18:1/24:2) | 6.15E-15 | | 2.49E-13 | -2.515 | 0.350 |
|  | 52944 | palmitoylcholine | 1.42E-13 | | 4.99E-12 | -0.143 | 0.025 |
|  | 53010 | lactosyl-N-palmitoyl-sphingosine (d18:1/16:0) | 3.35E-13 | | 1.10E-11 | -2.269 | 0.334 |
|  | 1125 | isoleucine | 1.27E-12 | | 3.92E-11 | 2.530 | 0.381 |
|  | 15676 | 3-methyl-2-oxovalerate | 3.01E-12 | | 8.78E-11 | 1.352 | 0.205 |
|  | 53261 | arachidonoylcholine | 1.14E-11 | | 3.15E-10 | -0.102 | 0.019 |
|  | 57370 | lactosyl-N-nervonoyl-sphingosine (d18:1/24:1) | 2.34E-11 | | 6.15E-10 | -1.265 | 0.205 |
|  | 40008 | isoleucylglycine | 1.04E-10 | | 2.61E-09 | -5.009 | 0.825 |
|  | 53260 | oleoylcholine | 1.70E-10 | | 4.07E-09 | -0.085 | 0.017 |
|  | 32506 | 2-linoleoylglycerol (18:2) | 7.73E-10 | | 1.77E-08 | -0.498 | 0.094 |
|  | 37506 | palmitoyl sphingomyelin (d18:1/16:0) | 9.53E-10 | | 2.09E-08 | -3.040 | 0.521 |
|  | 47154 | sphingomyelin (d18:2/14:0, d18:1/14:1) | 1.14E-09 | | 2.40E-08 | -0.664 | 0.118 |
|  | 53230 | 3-hydroxyhexanoate | 1.54E-09 | | 3.12E-08 | 1.021 | 0.182 |
|  | 52713 | 1-(1-enyl-palmitoyl)-2-palmitoleoyl-GPC (P-16:0/16:1) | 1.67E-09 | | 3.25E-08 | -1.697 | 0.297 |
|  | 1638 | arginine | 1.75E-09 | | 3.29E-08 | -2.790 | 0.484 |
|  | 35305 | 1-palmitoyl-GPI (16:0) | 1.86E-09 | | 3.38E-08 | -0.795 | 0.146 |
|  | 43802 | guanidinoacetate | 2.15E-09 | | 3.77E-08 | -1.706 | 0.298 |
|  | 32457 | 3-hydroxylaurate | 3.34E-09 | | 5.67E-08 | 0.782 | 0.138 |
|  | 43264 | 3-hydroxybutyrylcarnitine (1) | 3.86E-09 | | 6.35E-08 | 0.477 | 0.091 |
|  | 33961 | 1-stearoyl-GPC (18:0) | 4.05E-09 | | 6.45E-08 | -2.520 | 0.448 |
|  | 531 | 3-hydroxy-3-methylglutarate | 8.05E-09 | | 1.24E-07 | 0.817 | 0.152 |
|  | 52234 | glycosyl-N-stearoyl-sphingosine (d18:1/18:0) | 1.09E-08 | | 1.64E-07 | -0.926 | 0.173 |
|  | 52435 | sphingomyelin (d18:2/23:0, d18:1/23:1, d17:1/24:1) | 1.52E-08 | | 2.22E-07 | -0.735 | 0.139 |
|  | 1121 | margarate (17:0) | 3.96E-08 | | 5.63E-07 | 1.263 | 0.240 |
|  | 15720 | N-acetylglutamate | 4.67E-08 | | 6.47E-07 | 1.036 | 0.197 |
|  | 47153 | sphingomyelin (d18:1/24:1, d18:2/24:0) | 6.76E-08 | | 9.12E-07 | -1.145 | 0.230 |
|  | 22116 | 4-methyl-2-oxopentanoate | 6.99E-08 | | 9.20E-07 | 0.998 | 0.190 |
|  | 52464 | 1-palmitoyl-2-arachidonoyl-GPE (16:0/20:4) | 9.38E-08 | | 1.20E-06 | 0.509 | 0.098 |
|  | 42459 | sphingomyelin (d18:2/16:0, d18:1/16:1) | 1.19E-07 | | 1.46E-06 | -1.627 | 0.326 |
|  | 44526 | 3-methyl-2-oxobutyrate | 1.17E-07 | | 1.47E-06 | 0.939 | 0.181 |
|  | 52716 | 1-(1-enyl-palmitoyl)-2-palmitoyl-GPC (P-16:0/16:0) | 1.38E-07 | | 1.62E-06 | -1.963 | 0.392 |
|  | 57330 | lignoceroyl sphingomyelin (d18:1/24:0) | 1.37E-07 | | 1.64E-06 | -0.914 | 0.180 |
|  | 52682 | 1-(1-enyl-palmitoyl)-2-linoleoyl-GPC (P-16:0/18:2) | 1.44E-07 | | 1.65E-06 | -1.918 | 0.384 |
|  | 53013 | glycosyl-N-palmitoyl-sphingosine (d18:1/16:0) | 1.54E-07 | | 1.72E-06 | -1.047 | 0.211 |
|  | 52474 | 1-(1-enyl-palmitoyl)-GPC (P-16:0) | 1.61E-07 | | 1.76E-06 | -2.003 | 0.411 |
|  | 36602 | 1-oleoyl-GPI (18:1) | 1.88E-07 | | 2.02E-06 | -0.665 | 0.137 |
|  | 52447 | 1-stearoyl-2-arachidonoyl-GPE (18:0/20:4) | 2.07E-07 | | 2.18E-06 | 0.578 | 0.114 |
|  | 46142 | mannitol/sorbitol | 2.65E-07 | | 2.74E-06 | 0.205 | 0.051 |
|  | 34419 | 1-linoleoyl-GPC (18:2) | 2.97E-07 | | 3.01E-06 | -1.360 | 0.277 |
|  | 52603 | 1,2-dilinoleoyl-GPC (18:2/18:2) | 3.07E-07 | | 3.05E-06 | -1.205 | 0.249 |
|  | 52478 | 1-(1-enyl-palmitoyl)-2-oleoyl-GPC (P-16:0/18:1) | 4.78E-07 | | 4.66E-06 | -1.716 | 0.357 |
|  | 33949 | gamma-glutamylglycine | 5.64E-07 | | 5.39E-06 | -0.656 | 0.142 |
|  | 17805 | dihomo-linoleate (20:2n6) | 6.26E-07 | | 5.88E-06 | 0.825 | 0.171 |
|  | 52434 | palmitoyl dihydrosphingomyelin (d18:0/16:0) | 8.12E-07 | | 7.49E-06 | -1.447 | 0.305 |
|  | 33587 | eicosenoate (20:1) | 9.75E-07 | | 8.84E-06 | 0.771 | 0.163 |
|  | 1643 | fumarate | 1.77E-06 | | 1.58E-05 | 0.842 | 0.188 |
|  | 48493 | sphingomyelin (d18:1/22:1, d18:2/22:0, d16:1/24:1) | 2.19E-06 | | 1.92E-05 | -1.374 | 0.300 |
|  | 2132 | citrulline | 2.69E-06 | | 2.32E-05 | -1.371 | 0.303 |
|  | 43231 | 6-oxopiperidine-2-carboxylate | 3.13E-06 | | 2.65E-05 | 0.659 | 0.144 |
|  | 33972 | 10-nonadecenoate (19:1n9) | 3.74E-06 | | 3.13E-05 | 0.692 | 0.154 |
|  | 52616 | 1-palmitoyl-2-stearoyl-GPC (16:0/18:0) | 3.81E-06 | | 3.13E-05 | -1.260 | 0.286 |
|  | 6146 | 2-aminoadipate | 3.99E-06 | | 3.23E-05 | 0.559 | 0.134 |
|  | 36594 | 1-linoleoyl-GPI (18:2) | 4.10E-06 | | 3.26E-05 | -0.626 | 0.143 |
|  | 48255 | arabonate/xylonate | 4.54E-06 | | 3.56E-05 | 0.714 | 0.171 |
|  | 40173 | L-urobilin | 4.70E-06 | | 3.64E-05 | 0.028 | 0.007 |
|  | 1358 | stearate (18:0) | 5.09E-06 | | 3.88E-05 | 1.849 | 0.415 |
|  | 42420 | erythronate | 5.43E-06 | | 4.08E-05 | 1.366 | 0.337 |
|  | 1898 | proline | 6.47E-06 | | 4.72E-05 | 1.562 | 0.354 |
|  | 19263 | 1-palmitoyl-2-oleoyl-GPE (16:0/18:1) | 6.45E-06 | | 4.78E-05 | 0.263 | 0.061 |
|  | 53 | glutamine | 6.78E-06 | | 4.88E-05 | -1.412 | 0.322 |
|  | 57 | glutamate | 7.00E-06 | | 4.97E-05 | 2.477 | 0.567 |
|  | 54923 | beta-citrylglutamate | 8.16E-06 | | 5.72E-05 | 0.648 | 0.149 |
|  | 45095 | 2-methylbutyrylcarnitine (C5) | 9.95E-06 | | 6.88E-05 | 0.579 | 0.134 |
|  | 48429 | methyl-4-hydroxybenzoate sulfate | 1.02E-05 | | 6.99E-05 | -0.131 | 0.035 |
|  | 42374 | 2-aminobutyrate | 1.12E-05 | | 7.54E-05 | 0.818 | 0.189 |
|  | 18369 | gamma-glutamylleucine | 1.20E-05 | | 7.90E-05 | 1.152 | 0.271 |
|  | 63 | cholesterol | 1.19E-05 | | 7.90E-05 | -0.600 | 0.145 |
|  | 54805 | 3beta-hydroxy-5-cholestenoate | 1.26E-05 | | 8.17E-05 | -1.039 | 0.248 |
|  | 1336 | palmitate (16:0) | 1.34E-05 | | 8.58E-05 | 1.200 | 0.280 |
|  | 528 | alpha-ketoglutarate | 1.77E-05 | | 1.12E-04 | 2.798 | 0.691 |
|  | 1126 | alanine | 1.82E-05 | | 1.14E-04 | 1.318 | 0.312 |
|  | 53176 | 1-linoleoyl-2-linolenoyl-GPC (18:2/18:3) | 2.43E-05 | | 1.50E-04 | -0.380 | 0.098 |
|  | 32980 | adrenate (22:4n6) | 2.65E-05 | | 1.60E-04 | 0.552 | 0.138 |
|  | 48491 | sphingomyelin (d18:1/20:1, d18:2/20:0) | 2.64E-05 | | 1.62E-04 | -0.966 | 0.235 |
|  | 36747 | deoxycarnitine | 3.28E-05 | | 1.96E-04 | -1.250 | 0.310 |
|  | 48141 | 2-keto-3-deoxy-gluconate | 3.94E-05 | | 2.33E-04 | 0.187 | 0.048 |
|  | 48258 | 1-oleoyl-GPC (18:1) | 4.01E-05 | | 2.34E-04 | -1.119 | 0.280 |
|  | 40703 | prolylglycine | 4.38E-05 | | 2.53E-04 | 1.478 | 0.397 |
|  | 20693 | tartronate (hydroxymalonate) | 4.51E-05 | | 2.58E-04 | -0.991 | 0.248 |
|  | 52433 | sphingomyelin (d17:1/16:0, d18:1/15:0, d16:1/17:0) | 4.66E-05 | | 2.64E-04 | -0.884 | 0.223 |
|  | 52452 | 1-stearoyl-2-linoleoyl-GPC (18:0/18:2) | 4.84E-05 | | 2.65E-04 | -1.636 | 0.411 |
|  | 33955 | 1-palmitoyl-GPC (16:0) | 4.75E-05 | | 2.66E-04 | -2.021 | 0.508 |
|  | 46225 | pyroglutamine | 4.81E-05 | | 2.66E-04 | -0.471 | 0.121 |
|  | 58 | glycine | 6.31E-05 | | 3.42E-04 | -1.172 | 0.307 |
|  | 33230 | 1-palmitoleoyl-GPC (16:1) | 6.42E-05 | | 3.45E-04 | -0.533 | 0.142 |
|  | 52468 | 1-stearoyl-2-linoleoyl-GPI (18:0/18:2) | 6.70E-05 | | 3.56E-04 | -0.654 | 0.171 |
|  | 443 | aspartate | 7.47E-05 | | 3.93E-04 | 4.215 | 1.081 |
|  | 15506 | choline | 8.11E-05 | | 4.23E-04 | 1.475 | 0.381 |
|  | 43258 | acisoga | 8.36E-05 | | 4.31E-04 | 0.381 | 0.099 |
|  | 46144 | methyl glucopyranoside (alpha + beta) | 9.41E-05 | | 4.80E-04 | -0.206 | 0.060 |
|  | 54955 | linoleoyl-arachidonoyl-glycerol (18:2/20:4) [1] | 9.98E-05 | | 5.05E-04 | 0.259 | 0.071 |
|  | 52436 | tricosanoyl sphingomyelin (d18:1/23:0) | 1.09E-04 | | 5.44E-04 | -0.495 | 0.131 |
|  | 52285 | oleate/vaccenate (18:1) | 1.20E-04 | | 5.95E-04 | 0.680 | 0.179 |
|  | 542 | 3-hydroxybutyrate (BHBA) | 1.26E-04 | | 6.19E-04 | 0.408 | 0.122 |
|  | 40499 | 4-hydroxyglutamate | 1.66E-04 | | 8.06E-04 | 0.310 | 0.085 |
|  | 35114 | 7-methylguanine | 1.72E-04 | | 8.29E-04 | -1.171 | 0.323 |
|  | 37097 | tryptophan betaine | 1.86E-04 | | 8.92E-04 | -0.285 | 0.086 |
|  | 32504 | docosapentaenoate (n3 DPA; 22:5n3) | 2.29E-04 | | 1.09E-03 | 0.576 | 0.159 |
|  | 54907 | hexanoylglutamine | 2.83E-04 | | 1.33E-03 | 0.254 | 0.080 |
|  | 52710 | 1-linoleoyl-2-arachidonoyl-GPC (18:2/20:4n6) | 3.26E-04 | | 1.52E-03 | -0.647 | 0.185 |
|  | 57365 | myristoyl dihydrosphingomyelin (d18:0/14:0) | 3.42E-04 | | 1.58E-03 | -0.496 | 0.142 |
|  | 33364 | gamma-glutamylthreonine | 3.55E-04 | | 1.62E-03 | -1.113 | 0.318 |
|  | 43807 | bilirubin (Z,Z) | 3.60E-04 | | 1.63E-03 | -0.136 | 0.040 |
|  | 40473 | hydantoin-5-propionic acid | 4.40E-04 | | 1.98E-03 | 0.226 | 0.067 |
|  | 54956 | linoleoyl-arachidonoyl-glycerol (18:2/20:4) [2] | 4.72E-04 | | 2.11E-03 | 0.150 | 0.045 |
|  | 54 | tryptophan | 5.06E-04 | | 2.24E-03 | -1.235 | 0.361 |
|  | 15443 | glucuronate | 5.12E-04 | | 2.24E-03 | 0.482 | 0.147 |
|  | 52473 | gamma-tocopherol/beta-tocopherol | 5.56E-04 | | 2.42E-03 | 0.471 | 0.138 |
|  | 37203 | androstenediol (3beta,17beta) disulfate (2) | 6.14E-04 | | 2.63E-03 | 0.549 | 0.161 |
|  | 31912 | glycolithocholate | 6.10E-04 | | 2.63E-03 | 0.204 | 0.064 |
|  | 1564 | citrate | 6.26E-04 | | 2.66E-03 | 1.449 | 0.428 |
|  | 33971 | 10-heptadecenoate (17:1n7) | 6.39E-04 | | 2.69E-03 | 0.475 | 0.140 |
|  | 48460 | propyl 4-hydroxybenzoate sulfate | 7.24E-04 | | 3.02E-03 | -0.064 | 0.024 |
|  | 48492 | behenoyl sphingomyelin (d18:1/22:0) | 7.96E-04 | | 3.30E-03 | -0.768 | 0.234 |
|  | 1498 | N6,N6,N6-trimethyllysine | 8.36E-04 | | 3.44E-03 | -0.413 | 0.133 |
|  | 52467 | 1-palmitoyl-2-arachidonoyl-GPI (16:0/20:4) | 8.46E-04 | | 3.45E-03 | -0.256 | 0.080 |
|  | 15772 | ribitol | 1.03E-03 | | 4.17E-03 | 0.451 | 0.170 |
|  | 1105 | linoleate (18:2n6) | 1.20E-03 | | 4.81E-03 | 0.687 | 0.214 |
|  | 20699 | erythritol | 1.22E-03 | | 4.86E-03 | 0.345 | 0.115 |
|  | 15685 | 5-hydroxylysine | 1.28E-03 | | 5.08E-03 | 0.380 | 0.120 |
|  | 48341 | 1-dihomo-linolenylglycerol (20:3) | 1.30E-03 | | 5.11E-03 | -0.184 | 0.065 |
|  | 1567 | vanillylmandelate (VMA) | 1.45E-03 | | 5.60E-03 | -0.417 | 0.143 |
|  | 35136 | 5-methyluridine (ribothymidine) | 1.44E-03 | | 5.62E-03 | -1.079 | 0.343 |
|  | 1605 | ursodeoxycholate | 1.53E-03 | | 5.86E-03 | -0.086 | 0.030 |
|  | 33967 | N-acetylisoleucine | 1.69E-03 | | 6.43E-03 | 0.745 | 0.244 |
|  | 42449 | 1-palmitoyl-2-linoleoyl-GPE (16:0/18:2) | 1.84E-03 | | 6.97E-03 | 0.215 | 0.070 |
|  | 37529 | sphingomyelin (d18:1/18:1, d18:2/18:0) | 2.64E-03 | | 9.92E-03 | -0.729 | 0.245 |
|  | 32415 | docosadienoate (22:2n6) | 2.97E-03 | | 1.11E-02 | 0.517 | 0.176 |
|  | 42002 | lanthionine | 3.36E-03 | | 1.25E-02 | 0.279 | 0.104 |
|  | 46165 | 3-methyl catechol sulfate (1) | 3.44E-03 | | 1.26E-02 | 0.146 | 0.051 |
|  | 22163 | EDTA | 3.62E-03 | | 1.32E-02 | -0.977 | 0.346 |
|  | 1561 | alpha-tocopherol | 3.95E-03 | | 1.42E-02 | -0.473 | 0.167 |
|  | 34035 | linolenate [alpha or gamma; (18:3n3 or 6)] | 3.94E-03 | | 1.43E-02 | 0.482 | 0.169 |
|  | 39379 | glycoursodeoxycholate | 4.60E-03 | | 1.65E-02 | -0.170 | 0.067 |
|  | 19130 | 1,2-dipalmitoyl-GPC (16:0/16:0) | 5.00E-03 | | 1.78E-02 | -0.887 | 0.322 |
|  | 37186 | 5alpha-androstan-3alpha,17beta-diol monosulfate (1) | 5.46E-03 | | 1.93E-02 | 0.310 | 0.112 |
|  | 48407 | dopamine 3-O-sulfate | 5.50E-03 | | 1.93E-02 | 0.098 | 0.040 |
|  | 52446 | 1-stearoyl-2-linoleoyl-GPE (18:0/18:2) | 5.64E-03 | | 1.95E-02 | 0.210 | 0.077 |
|  | 35675 | 2-hydroxypalmitate | 5.62E-03 | | 1.96E-02 | -1.083 | 0.399 |
|  | 36808 | dimethylarginine (SDMA + ADMA) | 5.76E-03 | | 1.98E-02 | -0.903 | 0.337 |
|  | 38754 | gamma-carboxyglutamate | 5.84E-03 | | 1.99E-02 | 0.541 | 0.204 |
|  | 42463 | sphingomyelin (d18:1/14:0, d16:1/16:0) | 5.89E-03 | | 2.00E-02 | -0.605 | 0.225 |
|  | 33384 | salicyluric glucuronide | 6.87E-03 | | 2.32E-02 | 0.080 | 0.032 |
|  | 35678 | hexadecanedioate (C16-DC) | 6.94E-03 | | 2.32E-02 | 0.274 | 0.106 |
|  | 52689 | 1-(1-enyl-palmitoyl)-2-arachidonoyl-GPC (P-16:0/20:4) | 7.02E-03 | | 2.34E-02 | -0.818 | 0.307 |
|  | 52449 | 1-stearoyl-2-arachidonoyl-GPI (18:0/20:4) | 7.45E-03 | | 2.46E-02 | -0.470 | 0.178 |
|  | 48425 | phenylacetylcarnitine | 7.51E-03 | | 2.47E-02 | 0.082 | 0.032 |
|  | 1508 | pantothenate | 7.60E-03 | | 2.48E-02 | 0.233 | 0.088 |
|  | 36103 | p-cresol sulfate | 7.66E-03 | | 2.49E-02 | 0.169 | 0.065 |
|  | 22132 | alpha-hydroxyisocaproate | 8.47E-03 | | 2.73E-02 | 0.455 | 0.175 |
|  | 48187 | N-acetyltaurine | 8.62E-03 | | 2.77E-02 | -0.422 | 0.178 |
|  | 52495 | sphingomyelin (d18:1/21:0, d17:1/22:0, d16:1/23:0) | 8.73E-03 | | 2.78E-02 | -0.266 | 0.103 |
|  | 45951 | 1-linolenoyl-GPC (18:3) | 9.42E-03 | | 2.99E-02 | -0.236 | 0.093 |
|  | 1552 | erucate (22:1n9) | 9.57E-03 | | 3.01E-02 | 0.503 | 0.226 |
|  | 1365 | myristate (14:0) | 1.03E-02 | | 3.23E-02 | 0.466 | 0.182 |
|  | 32328 | hexanoylcarnitine (C6) | 1.06E-02 | | 3.30E-02 | 0.129 | 0.062 |
|  | 52983 | glycochenodeoxycholate glucuronide (1) | 1.08E-02 | | 3.33E-02 | 0.168 | 0.072 |
|  | 22137 | homoarginine | 1.12E-02 | | 3.44E-02 | -0.450 | 0.184 |
|  | 54961 | oleoyl-arachidonoyl-glycerol (18:1/20:4) [2] | 1.18E-02 | | 3.60E-02 | 0.126 | 0.053 |
|  | 52690 | 1-linoleoyl-GPA (18:2) | 1.46E-02 | | 4.42E-02 | 2.508 | 1.034 |
|  | 15990 | glycerophosphorylcholine (GPC) | 1.48E-02 | | 4.45E-02 | -0.809 | 0.343 |
|  | 43761 | 2-aminoheptanoate | 1.48E-02 | | 4.46E-02 | 0.340 | 0.141 |
|  | 35428 | tiglylcarnitine (C5:1-DC) | 1.49E-02 | | 4.47E-02 | 0.389 | 0.162 |
|  | 52726 | 1-stearoyl-2-oleoyl-GPI (18:0/18:1) | 1.55E-02 | | 4.60E-02 | -0.214 | 0.095 |
|  | 1587 | N-acetylleucine | 1.64E-02 | | 4.83E-02 | 0.517 | 0.226 |
|  | 32620 | glycolithocholate sulfate | 1.70E-02 | | 5.00E-02 | 0.175 | 0.074 |
| Non-Obesity | 20675 | 1,5-anhydroglucitol (1,5-AG) | 3.81E-15 | | 2.01E-12 | -3.460 | 0.545 |
| (n=221) | 48153 | mannose | 7.91E-14 | | 2.09E-11 | 3.788 | 0.669 |
|  | 53242 | 5-bromotryptophan | 6.33E-09 | | 1.11E-06 | -2.301 | 0.449 |
|  | 40008 | isoleucylglycine | 1.60E-08 | | 2.11E-06 | -10.045 | 2.110 |
|  | 52984 | 3-hydroxybutyrylcarnitine (2) | 7.10E-08 | | 7.48E-06 | 1.523 | 0.336 |
|  | 48195 | fructose | 1.41E-07 | | 1.24E-05 | 1.118 | 0.253 |
|  | 27731 | ribonate | 1.29E-06 | | 9.71E-05 | 1.642 | 0.427 |
|  | 52437 | sphingomyelin (d18:2/24:1, d18:1/24:2) | 1.62E-06 | | 1.06E-04 | -3.355 | 0.782 |
|  | 1303 | malate | 8.93E-06 | | 5.23E-04 | 2.670 | 0.708 |
|  | 52435 | sphingomyelin (d18:2/23:0, d18:1/23:1, d17:1/24:1) | 1.27E-05 | | 5.60E-04 | -1.282 | 0.327 |
|  | 52474 | 1-(1-enyl-palmitoyl)-GPC (P-16:0) | 1.22E-05 | | 5.86E-04 | -4.248 | 1.113 |
|  | 1643 | fumarate | 1.12E-05 | | 5.92E-04 | 1.652 | 0.417 |
|  | 47154 | sphingomyelin (d18:2/14:0, d18:1/14:1) | 1.55E-05 | | 6.28E-04 | -1.521 | 0.406 |
|  | 42459 | sphingomyelin (d18:2/16:0, d18:1/16:1) | 2.00E-05 | | 7.54E-04 | -3.066 | 0.804 |
|  | 22001 | 3-hydroxyoctanoate | 2.64E-05 | | 9.28E-04 | 0.823 | 0.242 |
|  | 1549 | 3-hydroxyisobutyrate | 2.87E-05 | | 9.45E-04 | 1.161 | 0.332 |
|  | 33961 | 1-stearoyl-GPC (18:0) | 4.09E-05 | | 1.27E-03 | -4.134 | 1.088 |
|  | 52616 | 1-palmitoyl-2-stearoyl-GPC (16:0/18:0) | 5.18E-05 | | 1.52E-03 | -2.612 | 0.726 |
|  | 52281 | 2-hydroxybutyrate/2-hydroxyisobutyrate | 7.49E-05 | | 2.08E-03 | 0.985 | 0.272 |
|  | 63 | cholesterol | 1.43E-04 | | 3.76E-03 | -1.236 | 0.360 |
|  | 52713 | 1-(1-enyl-palmitoyl)-2-palmitoleoyl-GPC (P-16:0/16:1) | 1.68E-04 | | 4.03E-03 | -2.146 | 0.630 |
|  | 15720 | N-acetylglutamate | 1.62E-04 | | 4.07E-03 | 1.603 | 0.446 |
|  | 22053 | 3-hydroxydecanoate | 2.05E-04 | | 4.70E-03 | 0.901 | 0.280 |
|  | 587 | gluconate | 2.47E-04 | | 5.20E-03 | 0.660 | 0.212 |
|  | 47153 | sphingomyelin (d18:1/24:1, d18:2/24:0) | 2.39E-04 | | 5.26E-03 | -1.791 | 0.566 |
|  | 12017 | 3-methoxytyrosine | 3.22E-04 | | 6.53E-03 | -1.903 | 0.591 |
|  | 57370 | lactosyl-N-nervonoyl-sphingosine (d18:1/24:1) | 4.04E-04 | | 7.89E-03 | -1.328 | 0.420 |
|  | 37506 | palmitoyl sphingomyelin (d18:1/16:0) | 4.53E-04 | | 8.53E-03 | -3.418 | 1.040 |
|  | 60 | leucine | 5.25E-04 | | 9.23E-03 | 2.647 | 0.798 |
|  | 15676 | 3-methyl-2-oxovalerate | 5.13E-04 | | 9.31E-03 | 1.268 | 0.387 |
|  | 53010 | lactosyl-N-palmitoyl-sphingosine (d18:1/16:0) | 5.75E-04 | | 9.78E-03 | -1.985 | 0.625 |
|  | 52433 | sphingomyelin (d17:1/16:0, d18:1/15:0, d16:1/17:0) | 5.98E-04 | | 9.85E-03 | -1.625 | 0.505 |
|  | 46142 | mannitol/sorbitol | 7.90E-04 | | 1.26E-02 | 0.424 | 0.151 |
|  | 43264 | 3-hydroxybutyrylcarnitine (1) | 8.55E-04 | | 1.33E-02 | 0.503 | 0.174 |
|  | 53176 | 1-linoleoyl-2-linolenoyl-GPC (18:2/18:3) | 9.56E-04 | | 1.44E-02 | -0.754 | 0.260 |
|  | 1638 | arginine | 1.03E-03 | | 1.46E-02 | -3.519 | 1.134 |
|  | 35305 | 1-palmitoyl-GPI (16:0) | 1.02E-03 | | 1.49E-02 | -0.901 | 0.308 |
|  | 53230 | 3-hydroxyhexanoate | 1.29E-03 | | 1.78E-02 | 1.098 | 0.361 |
|  | 52467 | 1-palmitoyl-2-arachidonoyl-GPI (16:0/20:4) | 1.34E-03 | | 1.82E-02 | -0.564 | 0.197 |
|  | 52716 | 1-(1-enyl-palmitoyl)-2-palmitoyl-GPC (P-16:0/16:0) | 1.52E-03 | | 2.01E-02 | -2.131 | 0.732 |
|  | 46165 | 3-methyl catechol sulfate (1) | 1.69E-03 | | 2.17E-02 | 0.232 | 0.078 |
|  | 43258 | acisoga | 1.77E-03 | | 2.22E-02 | 0.662 | 0.224 |
|  | 52478 | 1-(1-enyl-palmitoyl)-2-oleoyl-GPC (P-16:0/18:1) | 2.22E-03 | | 2.60E-02 | -2.001 | 0.702 |
|  | 57365 | myristoyl dihydrosphingomyelin (d18:0/14:0) | 2.28E-03 | | 2.61E-02 | -1.052 | 0.375 |
|  | 37203 | androstenediol (3beta,17beta) disulfate (2) | 2.15E-03 | | 2.64E-02 | 0.952 | 0.319 |
|  | 33949 | gamma-glutamylglycine | 2.22E-03 | | 2.65E-02 | -0.823 | 0.290 |
|  | 46225 | pyroglutamine | 2.39E-03 | | 2.68E-02 | -0.679 | 0.239 |
|  | 1126 | alanine | 2.45E-03 | | 2.69E-02 | 1.966 | 0.669 |
|  | 52449 | 1-stearoyl-2-arachidonoyl-GPI (18:0/20:4) | 2.80E-03 | | 3.01E-02 | -1.169 | 0.414 |
|  | 52436 | tricosanoyl sphingomyelin (d18:1/23:0) | 2.94E-03 | | 3.09E-02 | -0.805 | 0.284 |
|  | 32445 | 3-methylxanthine | 3.36E-03 | | 3.47E-02 | 0.200 | 0.073 |
|  | 52682 | 1-(1-enyl-palmitoyl)-2-linoleoyl-GPC (P-16:0/18:2) | 3.49E-03 | | 3.54E-02 | -1.940 | 0.716 |
|  | 15336 | tartarate | 3.84E-03 | | 3.82E-02 | 0.719 | 0.383 |
|  | 1125 | isoleucine | 4.00E-03 | | 3.91E-02 | 2.169 | 0.776 |
|  | 53 | glutamine | 4.32E-03 | | 4.14E-02 | -1.739 | 0.640 |
|  | 57330 | lignoceroyl sphingomyelin (d18:1/24:0) | 4.48E-03 | | 4.22E-02 | -0.970 | 0.358 |
|  | 48448 | 3-hydroxypyridine sulfate | 4.93E-03 | | 4.26E-02 | 0.111 | 0.040 |
|  | 15745 | methylsuccinate | 5.04E-03 | | 4.28E-02 | 0.623 | 0.228 |
|  | 20676 | maleate | 4.66E-03 | | 4.31E-02 | 0.358 | 0.145 |
|  | 52234 | glycosyl-N-stearoyl-sphingosine (d18:1/18:0) | 4.91E-03 | | 4.31E-02 | -0.872 | 0.338 |
|  | 35320 | catechol sulfate | 4.75E-03 | | 4.32E-02 | 0.195 | 0.071 |
|  | 22116 | 4-methyl-2-oxopentanoate | 4.91E-03 | | 4.38E-02 | 0.963 | 0.351 |
|  | 52434 | palmitoyl dihydrosphingomyelin (d18:0/16:0) | 5.82E-03 | | 4.87E-02 | -1.656 | 0.642 |
|  | 47114 | ferulic acid 4-sulfate | 6.02E-03 | | 4.96E-02 | 0.084 | 0.034 |
| Obesity only | 52944 | palmitoylcholine | 1.34E-11 | | 5.42E-10 | -0.149 | 0.029 |
| (n=584) | 53261 | arachidonoylcholine | 1.50E-10 | | 5.64E-09 | -0.112 | 0.023 |
|  | 32506 | 2-linoleoylglycerol (18:2) | 6.58E-09 | | 1.92E-07 | -0.601 | 0.119 |
|  | 53260 | oleoylcholine | 9.37E-09 | | 2.35E-07 | -0.088 | 0.020 |
|  | 32457 | 3-hydroxylaurate | 1.29E-08 | | 3.08E-07 | 0.899 | 0.167 |
|  | 52464 | 1-palmitoyl-2-arachidonoyl-GPE (16:0/20:4) | 1.64E-08 | | 3.75E-07 | 0.642 | 0.121 |
|  | 43802 | guanidinoacetate | 4.16E-08 | | 9.12E-07 | -1.931 | 0.368 |
|  | 531 | 3-hydroxy-3-methylglutarate | 1.32E-07 | | 2.48E-06 | 0.881 | 0.183 |
|  | 1121 | margarate (17:0) | 2.09E-07 | | 3.67E-06 | 1.435 | 0.293 |
|  | 52447 | 1-stearoyl-2-arachidonoyl-GPE (18:0/20:4) | 3.90E-07 | | 6.62E-06 | 0.653 | 0.134 |
|  | 17805 | dihomo-linoleate (20:2n6) | 5.48E-07 | | 9.01E-06 | 0.977 | 0.205 |
|  | 19263 | 1-palmitoyl-2-oleoyl-GPE (16:0/18:1) | 8.90E-07 | | 1.34E-05 | 0.356 | 0.078 |
|  | 33587 | eicosenoate (20:1) | 8.60E-07 | | 1.37E-05 | 0.939 | 0.202 |
|  | 33972 | 10-nonadecenoate (19:1n9) | 3.35E-06 | | 4.90E-05 | 0.813 | 0.184 |
|  | 1358 | stearate (18:0) | 4.08E-06 | | 5.80E-05 | 2.321 | 0.524 |
|  | 32980 | adrenate (22:4n6) | 4.65E-06 | | 6.27E-05 | 0.714 | 0.168 |
|  | 2132 | citrulline | 5.24E-06 | | 6.72E-05 | -1.573 | 0.359 |
|  | 44526 | 3-methyl-2-oxobutyrate | 5.17E-06 | | 6.80E-05 | 0.944 | 0.212 |
|  | 54956 | linoleoyl-arachidonoyl-glycerol (18:2/20:4) [2] | 6.01E-06 | | 7.53E-05 | 0.268 | 0.066 |
|  | 15772 | ribitol | 7.35E-06 | | 8.79E-05 | 1.137 | 0.268 |
|  | 1336 | palmitate (16:0) | 1.07E-05 | | 1.20E-04 | 1.432 | 0.335 |
|  | 54955 | linoleoyl-arachidonoyl-glycerol (18:2/20:4) [1] | 1.96E-05 | | 2.07E-04 | 0.389 | 0.096 |
|  | 53013 | glycosyl-N-palmitoyl-sphingosine (d18:1/16:0) | 2.61E-05 | | 2.69E-04 | -1.054 | 0.259 |
|  | 48255 | arabonate/xylonate | 2.74E-05 | | 2.77E-04 | 0.770 | 0.203 |
|  | 48493 | sphingomyelin (d18:1/22:1, d18:2/22:0, d16:1/24:1) | 2.86E-05 | | 2.84E-04 | -1.472 | 0.364 |
|  | 36602 | 1-oleoyl-GPI (18:1) | 4.28E-05 | | 4.10E-04 | -0.651 | 0.166 |
|  | 52603 | 1,2-dilinoleoyl-GPC (18:2/18:2) | 4.69E-05 | | 4.41E-04 | -1.170 | 0.300 |
|  | 36594 | 1-linoleoyl-GPI (18:2) | 4.86E-05 | | 4.48E-04 | -0.669 | 0.173 |
|  | 40173 | L-urobilin | 5.99E-05 | | 5.25E-04 | 0.032 | 0.010 |
|  | 52285 | oleate/vaccenate (18:1) | 6.60E-05 | | 5.43E-04 | 0.823 | 0.212 |
|  | 35114 | 7-methylguanine | 6.40E-05 | | 5.43E-04 | -1.500 | 0.391 |
|  | 528 | alpha-ketoglutarate | 6.71E-05 | | 5.43E-04 | 3.155 | 0.855 |
|  | 542 | 3-hydroxybutyrate (BHBA) | 7.89E-05 | | 6.10E-04 | 0.562 | 0.169 |
|  | 48429 | methyl-4-hydroxybenzoate sulfate | 7.83E-05 | | 6.15E-04 | -0.149 | 0.044 |
|  | 37097 | tryptophan betaine | 8.56E-05 | | 6.53E-04 | -0.366 | 0.111 |
|  | 1898 | proline | 1.05E-04 | | 7.91E-04 | 1.615 | 0.427 |
|  | 42374 | 2-aminobutyrate | 1.17E-04 | | 8.65E-04 | 0.868 | 0.230 |
|  | 43231 | 6-oxopiperidine-2-carboxylate | 1.19E-04 | | 8.67E-04 | 0.631 | 0.168 |
|  | 32504 | docosapentaenoate (n3 DPA; 22:5n3) | 1.24E-04 | | 8.91E-04 | 0.718 | 0.193 |
|  | 32415 | docosadienoate (22:2n6) | 1.44E-04 | | 9.96E-04 | 0.855 | 0.231 |
|  | 34419 | 1-linoleoyl-GPC (18:2) | 1.57E-04 | | 1.06E-03 | -1.180 | 0.324 |
|  | 54 | tryptophan | 1.61E-04 | | 1.07E-03 | -1.612 | 0.438 |
|  | 48491 | sphingomyelin (d18:1/20:1, d18:2/20:0) | 1.93E-04 | | 1.27E-03 | -1.025 | 0.280 |
|  | 42420 | erythronate | 1.99E-04 | | 1.29E-03 | 1.317 | 0.399 |
|  | 33971 | 10-heptadecenoate (17:1n7) | 2.24E-04 | | 1.42E-03 | 0.586 | 0.162 |
|  | 18369 | gamma-glutamylleucine | 2.31E-04 | | 1.44E-03 | 1.125 | 0.315 |
|  | 52473 | gamma-tocopherol/beta-tocopherol | 2.62E-04 | | 1.60E-03 | 0.607 | 0.169 |
|  | 52452 | 1-stearoyl-2-linoleoyl-GPC (18:0/18:2) | 3.04E-04 | | 1.84E-03 | -1.723 | 0.487 |
|  | 54923 | beta-citrylglutamate | 3.63E-04 | | 2.17E-03 | 0.582 | 0.167 |
|  | 54805 | 3beta-hydroxy-5-cholestenoate | 3.74E-04 | | 2.21E-03 | -1.147 | 0.333 |
|  | 33364 | gamma-glutamylthreonine | 3.96E-04 | | 2.31E-03 | -1.262 | 0.365 |
|  | 6146 | 2-aminoadipate | 4.29E-04 | | 2.48E-03 | 0.473 | 0.154 |
|  | 1564 | citrate | 4.85E-04 | | 2.77E-03 | 1.703 | 0.496 |
|  | 20693 | tartronate (hydroxymalonate) | 5.62E-04 | | 3.18E-03 | -0.969 | 0.286 |
|  | 1105 | linoleate (18:2n6) | 5.88E-04 | | 3.29E-03 | 0.842 | 0.248 |
|  | 48460 | propyl 4-hydroxybenzoate sulfate | 6.52E-04 | | 3.61E-03 | -0.084 | 0.032 |
|  | 54907 | hexanoylglutamine | 7.43E-04 | | 4.07E-03 | 0.285 | 0.099 |
|  | 57 | glutamate | 8.54E-04 | | 4.63E-03 | 2.191 | 0.674 |
|  | 42449 | 1-palmitoyl-2-linoleoyl-GPE (16:0/18:2) | 1.07E-03 | | 5.64E-03 | 0.257 | 0.081 |
|  | 36747 | deoxycarnitine | 1.26E-03 | | 6.46E-03 | -1.147 | 0.365 |
|  | 45095 | 2-methylbutyrylcarnitine (C5) | 1.26E-03 | | 6.48E-03 | 0.476 | 0.150 |
|  | 22137 | homoarginine | 1.45E-03 | | 7.25E-03 | -0.700 | 0.225 |
|  | 40703 | prolylglycine | 1.49E-03 | | 7.34E-03 | 1.391 | 0.466 |
|  | 48141 | 2-keto-3-deoxy-gluconate | 1.48E-03 | | 7.36E-03 | 0.158 | 0.053 |
|  | 15122 | glycerol | 1.58E-03 | | 7.69E-03 | 0.507 | 0.163 |
|  | 52468 | 1-stearoyl-2-linoleoyl-GPI (18:0/18:2) | 1.61E-03 | | 7.78E-03 | -0.615 | 0.199 |
|  | 22163 | EDTA | 1.83E-03 | | 8.76E-03 | -1.217 | 0.409 |
|  | 33230 | 1-palmitoleoyl-GPC (16:1) | 1.98E-03 | | 9.36E-03 | -0.504 | 0.168 |
|  | 48187 | N-acetyltaurine | 2.16E-03 | | 1.02E-02 | -0.708 | 0.240 |
|  | 40499 | 4-hydroxyglutamate | 2.59E-03 | | 1.21E-02 | 0.274 | 0.095 |
|  | 33955 | 1-palmitoyl-GPC (16:0) | 2.65E-03 | | 1.22E-02 | -1.767 | 0.598 |
|  | 34035 | linolenate [alpha or gamma; (18:3n3 or 6)] | 2.80E-03 | | 1.28E-02 | 0.567 | 0.194 |
|  | 443 | aspartate | 2.85E-03 | | 1.29E-02 | 3.554 | 1.208 |
|  | 54961 | oleoyl-arachidonoyl-glycerol (18:1/20:4) [2] | 2.90E-03 | | 1.30E-02 | 0.203 | 0.072 |
|  | 35628 | 1-oleoyl-GPE (18:1) | 3.11E-03 | | 1.39E-02 | 0.506 | 0.174 |
|  | 1365 | myristate (14:0) | 4.08E-03 | | 1.81E-02 | 0.602 | 0.212 |
|  | 54946 | oleoyl-oleoyl-glycerol (18:1/18:1) [2] | 4.26E-03 | | 1.87E-02 | 0.196 | 0.070 |
|  | 1567 | vanillylmandelate (VMA) | 4.42E-03 | | 1.91E-02 | -0.411 | 0.160 |
|  | 52446 | 1-stearoyl-2-linoleoyl-GPE (18:0/18:2) | 4.42E-03 | | 1.92E-02 | 0.253 | 0.091 |
|  | 37192 | 5alpha-androstan-3beta,17beta-diol monosulfate (2) | 4.98E-03 | | 2.09E-02 | 0.749 | 0.275 |
|  | 40473 | hydantoin-5-propionic acid | 5.19E-03 | | 2.16E-02 | 0.202 | 0.075 |
|  | 15506 | choline | 5.29E-03 | | 2.18E-02 | 1.182 | 0.430 |
|  | 58 | glycine | 5.28E-03 | | 2.19E-02 | -0.967 | 0.358 |
|  | 54958 | palmitoyl-arachidonoyl-glycerol (16:0/20:4) [2] | 5.64E-03 | | 2.28E-02 | 0.303 | 0.115 |
|  | 21184 | 1-oleoylglycerol (18:1) | 5.82E-03 | | 2.32E-02 | 0.491 | 0.192 |
|  | 48341 | 1-dihomo-linolenylglycerol (20:3) | 5.98E-03 | | 2.36E-02 | -0.191 | 0.075 |
|  | 52710 | 1-linoleoyl-2-arachidonoyl-GPC (18:2/20:4n6) | 6.07E-03 | | 2.38E-02 | -0.574 | 0.214 |
|  | 1498 | N6,N6,N6-trimethyllysine | 6.12E-03 | | 2.38E-02 | -0.397 | 0.150 |
|  | 15443 | glucuronate | 6.22E-03 | | 2.39E-02 | 0.444 | 0.171 |
|  | 44876 | gamma-CEHC | 6.21E-03 | | 2.40E-02 | -0.221 | 0.084 |
|  | 1561 | alpha-tocopherol | 6.60E-03 | | 2.52E-02 | -0.513 | 0.192 |
|  | 36746 | 2-hydroxy-3-methylvalerate | 7.30E-03 | | 2.70E-02 | 0.420 | 0.164 |
|  | 32391 | 1,3-dimethylurate | 7.21E-03 | | 2.71E-02 | -0.037 | 0.021 |
|  | 606 | uridine | 7.37E-03 | | 2.71E-02 | -0.879 | 0.332 |
|  | 46144 | methyl glucopyranoside (alpha + beta) | 7.69E-03 | | 2.81E-02 | -0.163 | 0.067 |
|  | 31912 | glycolithocholate | 8.32E-03 | | 3.02E-02 | 0.192 | 0.079 |
|  | 12129 | beta-hydroxyisovalerate | 9.50E-03 | | 3.42E-02 | 0.793 | 0.309 |
|  | 35428 | tiglylcarnitine (C5:1-DC) | 9.91E-03 | | 3.52E-02 | 0.495 | 0.197 |
|  | 34389 | 1-methylxanthine | 1.03E-02 | | 3.60E-02 | -0.122 | 0.048 |
|  | 38768 | 15-methylpalmitate (i17:0) | 1.02E-02 | | 3.60E-02 | 0.614 | 0.243 |
|  | 22132 | alpha-hydroxyisocaproate | 1.08E-02 | | 3.71E-02 | 0.544 | 0.216 |
|  | 38754 | gamma-carboxyglutamate | 1.07E-02 | | 3.71E-02 | 0.564 | 0.232 |
|  | 46223 | linoleoylcarnitine (C18:2) | 1.08E-02 | | 3.73E-02 | -0.508 | 0.202 |
|  | 54945 | oleoyl-oleoyl-glycerol (18:1/18:1) [1] | 1.13E-02 | | 3.86E-02 | 0.248 | 0.101 |
|  | 35186 | 1-arachidonoyl-GPE (20:4n6) | 1.14E-02 | | 3.87E-02 | 0.557 | 0.222 |
|  | 37529 | sphingomyelin (d18:1/18:1, d18:2/18:0) | 1.19E-02 | | 4.02E-02 | -0.712 | 0.286 |
|  | 48492 | behenoyl sphingomyelin (d18:1/22:0) | 1.25E-02 | | 4.10E-02 | -0.674 | 0.274 |
|  | 33384 | salicyluric glucuronide | 1.25E-02 | | 4.10E-02 | 0.098 | 0.042 |
|  | 36808 | dimethylarginine (SDMA + ADMA) | 1.24E-02 | | 4.12E-02 | -0.943 | 0.391 |
|  | 15685 | 5-hydroxylysine | 1.24E-02 | | 4.14E-02 | 0.343 | 0.140 |
|  | 48407 | dopamine 3-O-sulfate | 1.29E-02 | | 4.19E-02 | 0.104 | 0.048 |
|  | 43807 | bilirubin (Z,Z) | 1.30E-02 | | 4.19E-02 | -0.112 | 0.047 |
|  | 52983 | glycochenodeoxycholate glucuronide (1) | 1.36E-02 | | 4.35E-02 | 0.228 | 0.113 |
|  | 32620 | glycolithocholate sulfate | 1.42E-02 | | 4.48E-02 | 0.233 | 0.097 |
|  | 20699 | erythritol | 1.41E-02 | | 4.48E-02 | 0.303 | 0.131 |
|  | 35678 | hexadecanedioate (C16-DC) | 1.44E-02 | | 4.48E-02 | 0.346 | 0.145 |
|  | 52431 | 1-palmitoleoylglycerol (16:1) | 1.41E-02 | | 4.50E-02 | 0.215 | 0.096 |
|  | 36103 | p-cresol sulfate | 1.57E-02 | | 4.85E-02 | 0.185 | 0.080 |
|  | 33936 | octanoylcarnitine (C8) | 1.59E-02 | | 4.89E-02 | 0.119 | 0.061 |
|  | 34445 | sphingosine 1-phosphate | 1.61E-02 | | 4.94E-02 | -0.455 | 0.194 |
|  |  |  |  | |  |  |  |

| **Table Supplemental Table 3. Top biological pathways, bioprocesses and chemical classes enriched in the obesity-T2D and nonobese-T2D comparisons** | | | |
| --- | --- | --- | --- |
| **Entities enriched in and unique to obese** |  |  |  |
| ***Chemical classification*** | *P value* | *Metabolites* | *HMDB identifiers* |
| **fatty acids and conjugates** | 1.09E-14 | various | HMDB02259 HMDB00220 HMDB00827 HMDB00806 HMDB05060 HMDB00746 HMDB00387 HMDB06528 HMDB02226 HMDB02231 HMDB60038 HMDB13622 HMDB00317 HMDB01161 HMDB00019 HMDB00207 HMDB03231 |
| **unsaturated fatty acids** | 2.14E-07 | various | HMDB05060 HMDB06528 HMDB02226 HMDB02231 HMDB13622 HMDB00207 HMDB03231 |
| **acyl carnitines** | 1.12E-04 | octanoylcarnitine; tiglylcarnitine; 2-methylbutyrylcarnitine; linoleoylcarnitine |  |
| ***Pathways*** |  |  |  |
| **Biosynthesis of unsaturated fatty acids** | 3.96E-07 | linoleate; palmitate; stearate; eicosadienoate; docosapentaenoate (22n-3); adrenate; 11Z-eicosenoate; gamma-linolenate; oleate |  |
| **Alpha Linolenic Acid and Linoleic Acid Metabolism** | 1.22E-02 | linoleate; docosapentaenoate (22n-3); adrenate; gamma-linolenate |  |
| **Carnitine Synthesis** | 1.22E-02 | glycine; oxoglutarate; N6,N6,N6-trimethyl-L-lysine; 4-trimethylammoniobutanoate |  |
| **Malate-Aspartate Shuttle** | 1.22E-02 | glutamate; aspartate; oxoglutarate |  |
| **Glutamate biosynthesis, oxoglutarate => glutamate** | 1.35E-02 | glutamate; oxoglutarate |  |
| **Linoleic acid metabolism, phospholipid => linoleate** | 1.35E-02 | linoleate; PC(18:2/20:4) |  |
| **phosphatidylcholine biosynthesis, choline => PC** | 3.92E-02 | choline; PC(18:2/20:4) |  |
| **eNOS activation** | 2.64E-02 | dimethylarginine (SDMA + ADMA); citrulline; palmitate |  |
| **Entities enriched in and unique to nonobese** |  |  |  |
|  |  |  |  |
| ***Chemical classification*** |  |  |  |
| **phosphatidylinositols** | 0.0118 | PI(18:0/20:4); PI(16:0/20:4) |  |
| **lipids**** | 0.0216 | none: de-enrichment |  |
|  |  |  |  |
| ***Pathways*** |  |  |  |
| **Glyoxylate and dicarboxylate metabolism** | 0.0485 | tartrate |  |
| **Nicotinate and nicotinamide metabolism** | 0.0485 | maleate |  |
| **P-values are FDR-corrected, as described in Methods** |  |  |  |
| **HMDB (Human Metabolomics Database identifiers used in the analysis, see text for details)** |  |  |  |
| **** classification identified with LipidMaps** |  |  |  |

| **Supplemental Table 4. 24 metabolites that are associated with type 2 diabetes unique to obesity and were associated with type 2 diabetes incidence at the 5-year follow-up.** | | | | | | |
| --- | --- | --- | --- | --- | --- | --- |
| CompID | BIOCHEMICAL | SUPER PATHWAY | SUB PATHWAY | P-Value | Beta | SE |
| 443 | aspartate | Amino Acid | Alanine and Aspartate Metabolism | 2.10E-03 | 6.240 | 1.991 |
| 57 | glutamate | Amino Acid | Glutamate Metabolism | 3.96E-03 | 2.705 | 0.933 |
| 38754 | gamma-carboxyglutamate | Amino Acid | Glutamate Metabolism | 7.96E-03 | 1.137 | 0.440 |
| 35428 | tiglylcarnitine (C5:1-DC) | Amino Acid | Leucine, Isoleucine and Valine Metabolism | 4.09E-02 | 0.625 | 0.301 |
| 44526 | 3-methyl-2-oxobutyrate | Amino Acid | Leucine, Isoleucine and Valine Metabolism | 9.39E-05 | 1.362 | 0.354 |
| 1898 | proline | Amino Acid | Urea cycle; Arginine and Proline Metabolism | 4.15E-02 | 1.349 | 0.653 |
| 18369 | gamma-glutamylleucine | Peptide | Gamma-glutamyl Amino Acid | 1.12E-03 | 1.660 | 0.503 |
| 528 | alpha-ketoglutarate | Energy | TCA Cycle | 4.65E-02 | 2.829 | 1.391 |
| 1365 | myristate (14:0) | Lipid | Long Chain Fatty Acid | 5.47E-03 | 1.036 | 0.370 |
| 1336 | palmitate (16:0) | Lipid | Long Chain Fatty Acid | 2.04E-03 | 1.717 | 0.561 |
| 1121 | margarate (17:0) | Lipid | Long Chain Fatty Acid | 4.54E-03 | 1.386 | 0.487 |
| 33971 | 10-heptadecenoate (17:1n7) | Lipid | Long Chain Fatty Acid | 2.94E-02 | 0.625 | 0.283 |
| 1358 | stearate (18:0) | Lipid | Long Chain Fatty Acid | 2.89E-03 | 2.437 | 0.818 |
| 33972 | 10-nonadecenoate (19:1n9) | Lipid | Long Chain Fatty Acid | 2.05E-02 | 0.709 | 0.304 |
| 32980 | adrenate (22:4n6) | Lipid | Polyunsaturated Fatty Acid (n3 and n6) | 1.46E-02 | 0.628 | 0.250 |
| 38768 | 15-methylpalmitate (i17:0) | Lipid | Fatty Acid, Branched | 2.13E-02 | 0.942 | 0.402 |
| 19263 | 1-palmitoyl-2-oleoyl-GPE (16:0/18:1) | Lipid | Phosphatidylethanolamine (PE) | 5.52E-03 | 0.285 | 0.099 |
| 42449 | 1-palmitoyl-2-linoleoyl-GPE (16:0/18:2) | Lipid | Phosphatidylethanolamine (PE) | 8.10E-03 | 0.365 | 0.135 |
| 52464 | 1-palmitoyl-2-arachidonoyl-GPE (16:0/20:4) | Lipid | Phosphatidylethanolamine (PE) | 3.89E-03 | 0.507 | 0.172 |
| 52446 | 1-stearoyl-2-linoleoyl-GPE (18:0/18:2) | Lipid | Phosphatidylethanolamine (PE) | 1.78E-02 | 0.347 | 0.144 |
| 52447 | 1-stearoyl-2-arachidonoyl-GPE (18:0/20:4) | Lipid | Phosphatidylethanolamine (PE) | 1.15E-02 | 0.529 | 0.207 |
| 35186 | 1-arachidonoyl-GPE (20:4n6) | Lipid | Lysophospholipid | 2.15E-02 | 0.751 | 0.321 |
| 48491 | sphingomyelin (d18:1/20:1, d18:2/20:0) | Lipid | Sphingomyelins | 4.26E-02 | -0.876 | 0.441 |
| 52473 | gamma-tocopherol/beta-tocopherol | Cofactors and Vitamins | Tocopherol Metabolism | 2.80E-02 | 0.572 | 0.259 |
|  |  |  |  |  |  |  |


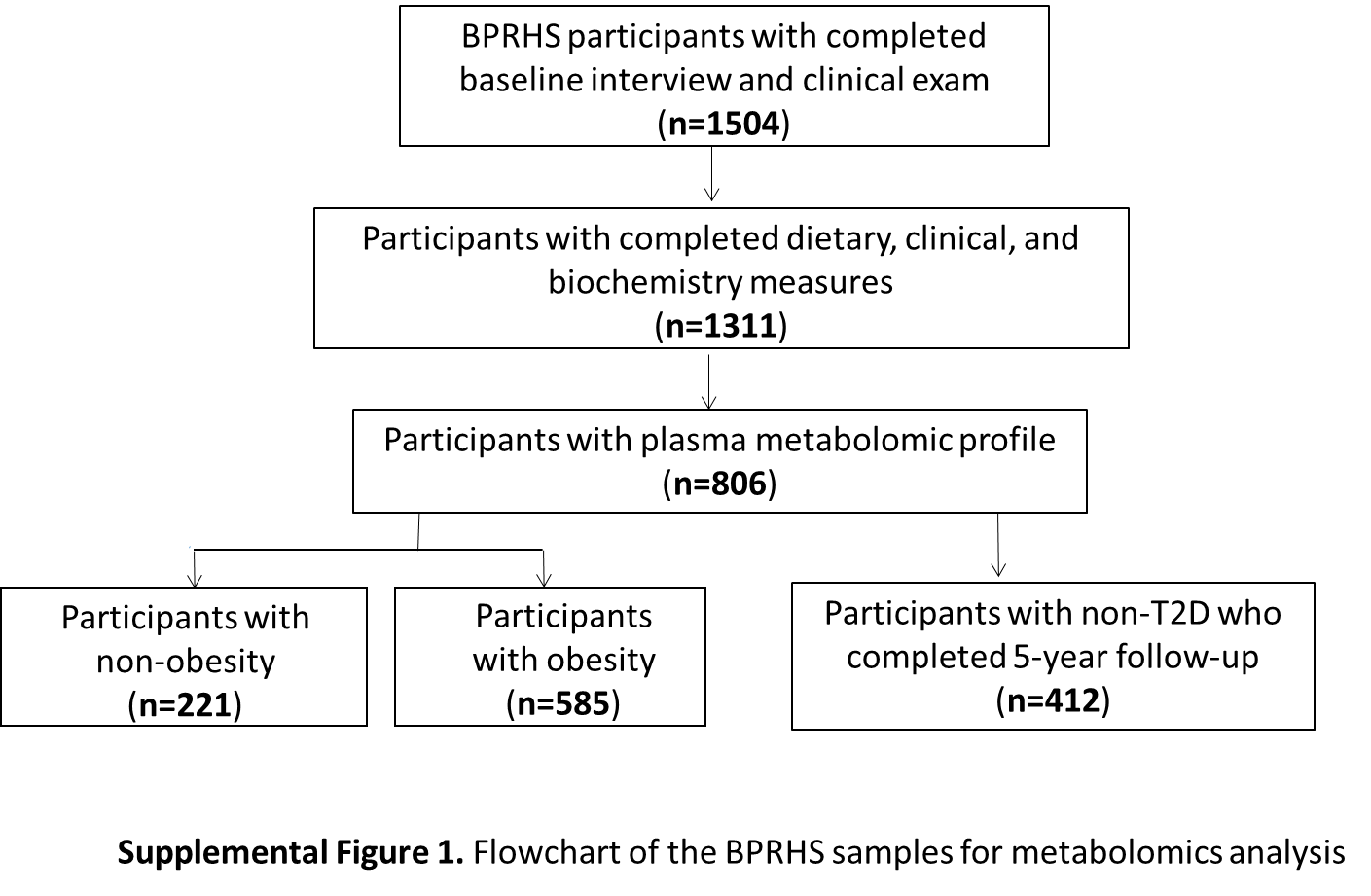

Supplement: Supplementary file 1 — Supplementary file1 (DOCX 112 kb) [file 11306_2021_1835_MOESM1_ESM.docx]
